# Collision-Induced Dissociation Studies of Synthetic Opioids for Non-targeted Analysis

**DOI:** 10.3389/fchem.2019.00331

**Published:** 2019-05-14

**Authors:** Joshua Klingberg, Adam Cawley, Ronald Shimmon, Shanlin Fu

**Affiliations:** ^1^Centre for Forensic Science, University of Technology Sydney, Ultimo, NSW, Australia; ^2^Australian Racing Forensic Laboratory, Racing NSW, Sydney, NSW, Australia

**Keywords:** synthetic opioids, collision-induced dissociation, non-targeted analysis, high-resolution mass spectrometry (HRMS), new psychoactive substances (NPS)

## Abstract

The continual introduction of a large number of new psychoactive substances, along with the large turnover of these substances, necessitates the development of non-targeted detection strategies to keep pace with the ever-changing drug market. The production of certified reference materials often lags behind the introduction of new substances to the market, therefore these detection strategies need to be able to function without relying on reference materials or library spectra. Synthetic opioids have recently emerged as a drug class of particular concern due to the health issues caused by their incredibly high potency. A common method which has been used for non-targeted analysis in the past involves the identification of common product ions formed as a result of the fragmentation of the parent molecule. These common fragments can then potentially be used as markers to indicate the presence of a particular class of compounds within a sample. In this study, standards of a number of different synthetic opioids, including 14 fentanyl derivatives, 7 AH series opioids, 4 U series opioids, 4 W series opioids and MT-45, were subjected to collision-induced dissociation studies to determine how the compounds fragment. The spectra obtained from these studies included a number of diagnostic fragments common to the different opioid classes that, when used in combination, show potential for use as class predictors. By using simple data processing techniques, such as extracted ion chromatograms, these diagnostic product ions identified can be applied to a non-targeted screening workflow.

## Introduction

**Figure 1:**
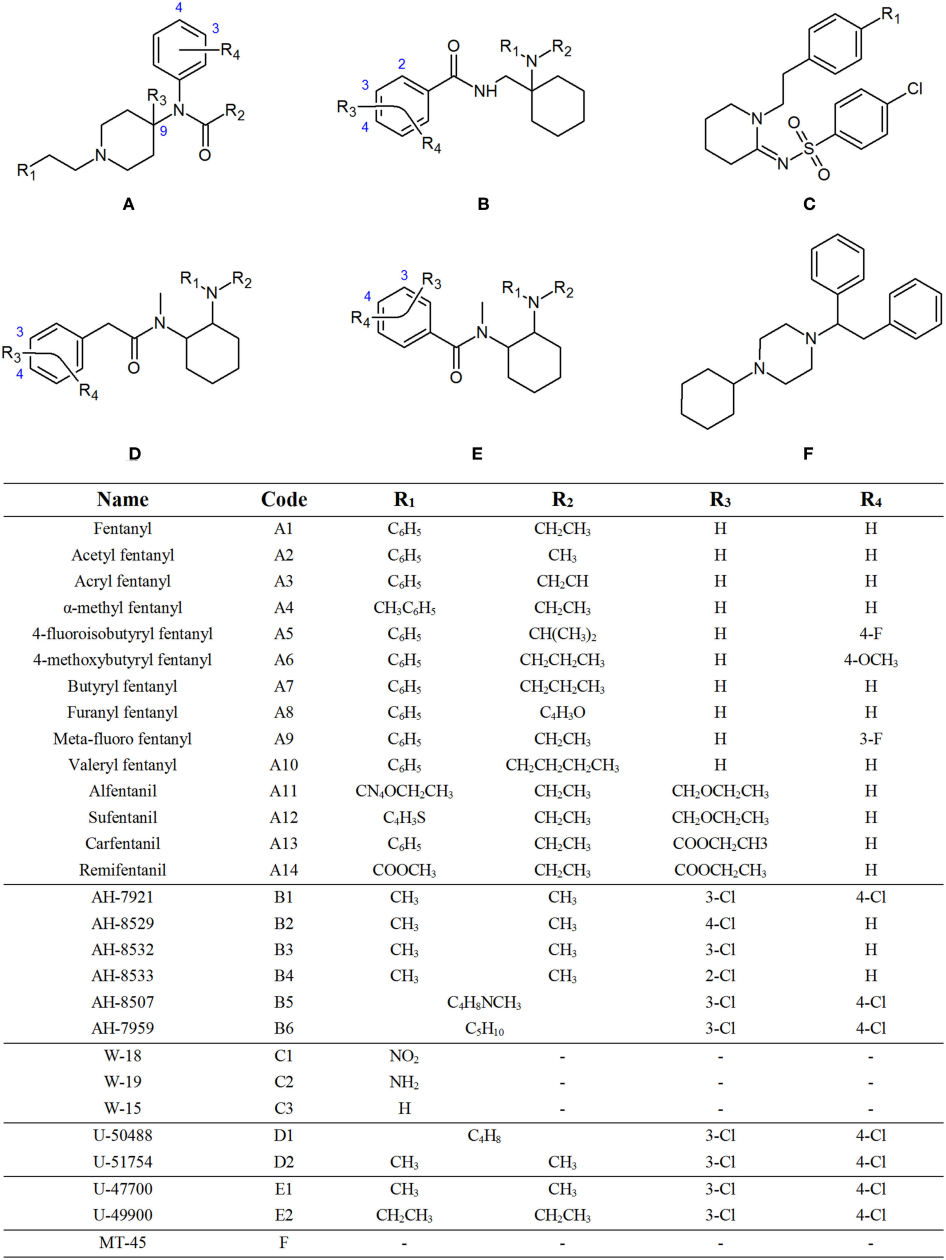
General chemical structure of fentanyls **(A)**, AH series opioids **(B)**, W series opioids **(C)**, U series opioids with **(D)**, and without **(E)** a methylene spacer and MT-45 **(F)**.

The term “opioid” literally means “opium-like substance.” Opium is the dried juice of the opium poppy, *Papaver Somniferum*, which has been used for the relief of pain for thousands of years (Ballantyne and LaForge, 2007). The actions of the opiates mirrors the actions of endogenous opioid peptides, such as endorphins, enkephalins and dynorphins, which exist within the central and peripheral nervous system as neurotransmitters and neuromodulators (Holden et al., 2005). Synthetic opioids are man-made compounds that act on the opioid receptors in the brain and mimic the effects of natural opiates, such as morphine and codeine (World Health Organisation, 2011). While there are a number of synthetic opioids known to forensic toxicologists, such as methadone, levomethorphan and pethidine, two groups have recently come under increased scrutiny, namely non-pharmaceutical fentanyls (NPFs), which are fentanyl derivatives that are obtained or synthesized illicitly, and novel synthetic opioids (NSOs). While there can be some overlap between these classifications, NSOs usually refer to the newer classes of opioids, such as the AH, U and W series', which were originally developed to act as new opioid agonists, but never brought to the market for human use (Lucyk and Nelson, 2017). Many of these compounds have recently emerged onto the illicit drug market. The United Nations Office on Drugs and Crime (UNODC) have identified opioids as the class of compounds which cause the most harm, accounting for 76% of all deaths attributed to drug use worldwide in 2016, with fentanyl derivatives alone accounting for 30% of all deaths in the United States (United Nations Office on Drugs Crime, 2018). In addition, there is an increasing trend of fentanyl derivatives, and other synthetic opioids, being mixed into illicit drugs, or being sold as prescription opioids. Figure 1 shows the chemical structures of the synthetic opioids investigated in this study grouped into subclasses based on their structural features. Individual structures for each of these opioids can be found in Figures S1–S4.

The continuous introduction of a large number of new psychoactive substances (NPS), along with the lack of certified reference materials (CRMs) for many of these compounds, highlights the inadequacy of traditional targeted screening methods to detect these substances (Pasin et al., 2017b). In particular, many synthetic opioids may not be detected by traditional approaches to illicit drug analysis, and many routine analyses do not screen for these compounds (Breindahl et al., 2017). This may be due to numerous factors, including a lack of cross-reactivity with traditional immunoassay screening methods and data for these compounds not being available in the mass spectral libraries that are used for drug screening (Mohr et al., 2016). To combat this, the aim of non-targeted analysis is to identify compounds from samples where the molecular content is unknown (Knolhoff and Croley, 2016). Since illicit drug seizures are, by their very nature, unknown entities, this approach to their analysis provides some obvious advantages.

The use of high-resolution mass spectrometry (HRMS) has been investigated for non-targeted analysis due to its ability to be operated in data-independent acquisition (DIA) modes in addition to traditional data-dependent acquisition (DDA) modes (Remane et al., 2016; Pasin et al., 2017b). While DIA has been shown to be capable of detecting more compounds at lower concentration than DDA (Arnhard et al., 2015), a challenge arises due to the production of “chimeric spectra.” DIA methods fragment all precursor ions simultaneously, producing a spectrum that includes product ions from all precursor ion subjected to CID (Oberacher and Arnhard, 2016; Pasin et al., 2017b). This means that the software cannot associate product ions with their correct precursor ions. It has been suggested that deconvolution algorithms may be used to exploit the full potential of DIA data (Oberacher and Arnhard, 2016), however the use of DDA may be useful in situation where rapid identification of product ions related to specific precursor ions is required.

The main advantage of HRMS over other mass spectrometric methods is its ability to differentiate between ions and losses that have the same nominal mass. For example, the loss of an NH_3_ molecule or an –OH group would equate to the same nominal mass change of 17 Da. This change may be indistinguishable using lower resolution MS techniques. If an MS technique with a high enough mass resolution was used, however, the accurate masses for those fragments of 17.0265 and 17.0027 Da respectively could be determined and therefore the losses differentiated. The effectiveness of collision-induced dissociation (CID) data for the development of non-targeted detection strategies has been previously demonstrated for different drug classes (Pasin et al., 2017a).

While fragmentation patterns have been proposed in the literature for various individual opioids, mainly fentanyl derivatives (Ohta et al., 1999; Lurie and Iio, 2009; Rittgen et al., 2012; Kronstrand et al., 2014; Patton et al., 2014; Breindahl et al., 2017; Fabregat-Safont et al., 2017; Helander et al., 2017; Rojkiewicz et al., 2017), there is a need for an overview of the CID pathways for all synthetic opioids to facilitate broad-spectrum opioid screening. Noble et al. (2017) previously investigated the use of HRMS data collected in DIA mode to identify 50 fentanyl analogs without the use of CRMs. In this study, the identification of common CID pathways is expanded to include both NPFs and NSOs. Additionally, the application of this information to non-targeted screening methods will be discussed.

## Materials and Methods

### Solvents and Reagents

All solvents used were liquid chromatography-mass spectrometry (LC-MS) grade. Acetonitrile, ethyl acetate and methanol were obtained from Merck (Darmstadt, Germany). Ammonium acetate and trichloroacetic acid were obtained from Sigma-Aldrich (Castle Hill, Australia). Acetic acid was obtained from Ajax Chemicals (Sydney, Australia). Ultrapure-grade water was obtained from a Smart2Pure ultra-pure water system (Thermo Scientific, Langenselbold, Hungary).

### Opioids Standards

Fentanyl citrate was purchased from Sigma-Aldrich (Castle Hill, NSW). Hydrochloride salts of acetyl fentanyl, 4-fluoroisobutyryl fentanyl, meta-fluoro fentanyl, AH-7921, AH-8529, AH-8533, U-51754, and W-19, along with neat solids of AH-7959, AH-8532, AH-8507, U-49900, and U-50488, were purchased from Sapphire Bioscience (Redfern, NSW). Hydrochloride salts of furanyl fentanyl, acryl fentanyl, 4-methoxybutyryl fentanyl, valeryl fentanyl, butyryl fentanyl, U-47700, and W-18, along with a neat solid of W-15 and MT-45 dihydrochloride hydrate, were purchased from Chiron Chemicals (Hawthorn, VIC). Remifentanil hydrochloride was purchased from GlaxoSmithKline (Boronia, VIC). Citrate salts of carfentanil, sufentanil and α-methyl fentanyl, along with alfentanil hydrochloride, were purchased from Janssen Pharmaceuticals (North Ryde, NSW).

### Sample Preparation

Drug standards were obtained as methanolic standards of varying concentrations. All opioid samples for the CID study were diluted in methanol to a concentration of 10 μg/mL. Ten microliter of each solution was evaporated to dryness under nitrogen, before being reconstituted in methanol and a 10 mM ammonium acetate (pH 4) buffer to give a final concentration of 1 μg/mL for analysis. All samples were refrigerated until analysis.

A spiked sample of equine plasma was prepared to test the application of the identified diagnostic product ions to a non-targeted screening workflow. A mixed standard containing fentanyl, acetyl fentanyl, AH-7921 and U-50488 was made with a concentration of 200 ng/mL in methanol. Two mL of blank equine plasma was spiked with the mixed standard to give concentrations ranging from 10 to 0.01 ng/mL. A blank sample was also prepared for extraction. The proteins were precipitated out of the samples by the addition of 200 μL of trichloroacetic acid (10% in H_2_O) and the pH of the samples was adjusted to 3–3.5 using hydrochloric acid. The samples were then centrifuged at 1,500 g for 15 min. Following centrifugation, the samples were extracted using XTRACKT® Gravity Flow DAU Extraction Columns (UCT Inc., Bristol, United States). The cartridges were first conditioned with 3 mL of methanol, followed by 3 mL of purified water. The samples were then loaded and the cartridges washed with 3 mL of 0.1 M acetic acid, before being dried under positive pressure. Three mL of methanol was run through the cartridges, which were dried again under positive pressure. Finally, the samples were eluted using 3 mL of an elution solvent containing 3% ammonia and 0.5% methanol in ethyl acetate.

One drop of 0.1 M methanolic hydrochloric acid was added to the samples before the solvent was evaporated under nitrogen at 60°C. The samples were then reconstituted with one drop of methanol and 100 μL of an ammonium acetate buffer (pH 3.9), before being transferred to a vial for analysis.

### Instrumental Analysis

Chromatographic separation was achieved using an Agilent Technologies (Santa Clara, CA, USA) 1290 Infinity II UHPLC, consisting of a high speed pump (G7120A), multisampler (G7167B) and thermostat and column compartment (G1316A, 35°C) coupled to an Agilent 6545 quadrupole time-of-flight mass spectrometer (QTOF-MS). All data acquisition was conducted using Agilent MassHunter Workstation (Version B.06.01). A sample volume of 5 μL was injected onto a Phenomenex (Torrance, CA, USA) Gemini 110 Å C18 LC column (2 × 50 mm, 5 μm particle size) using a gradient elution method with a flow rate of 0.5 mL/min and a total analysis time of 11.5 min. Mobile phase A consisted of a 10 mM ammonium acetate (pH 9) buffer and mobile phase B consisted of 0.1% acetic acid in acetonitrile. Initial mobile phase composition was 99% A, which was held for 2 min before being decreased linearly to 20% A over 6.5 min. The mobile phase was then returned to 99% A over 3 min.

The QTOF-MS was operated in positive electrospray ionization mode (ESI+) with capillary and fragmentor voltages of 3,500 and 100 V, respectively. An Auto-MS/MS (data-dependent) acquisition mode was used with mass ranges of 100–1,000 m/z for MS and 50–500 m/z for MS/MS and a scan rate of 10 spectra/s for both. A preferred precursor ion table was populated with the corresponding [M + H]^+^ values for each opioid standard and the acquisition method was set to use the preferred list only for MS/MS analysis. CID experiments were performed using collision energies (CE) of 10, 20 and 40 eV in separate experiments, with nitrogen as the collision gas.

For the spiked plasma samples, the QTOF was operated in ESI+ mode with capillary and fragmentor voltages of 3,500 and 100 V, respectively. An AllIonsMS (DIA) data acquisition mode was used with a mass range of 35–1,000 m/z. Spectra were obtained with an acquisition rate of 10 spectra/s and CEs of 10, 20, and 40 eV were used for CID.

All data acquired was processed using Agilent MassHunter Qualitative Analysis Software (Version B.07.00). The Find by Auto-MS/MS function was used to obtain MS/MS spectra for all precursor ions selected for CID experiments. All MS/MS spectra obtained from the analyzed standards can be found in the supplementary material (Figures S5–S31). For the spiked plasma samples, extracted ion chromatograms (EIC or XIC) were created for a number of diagnostic product ions identified from the CID study to screen for possible compounds present within the samples.

## Results and Discussion

### Collision-Induced Dissociation Pathways

#### Fentanyl Derivatives

**Figure 2:**
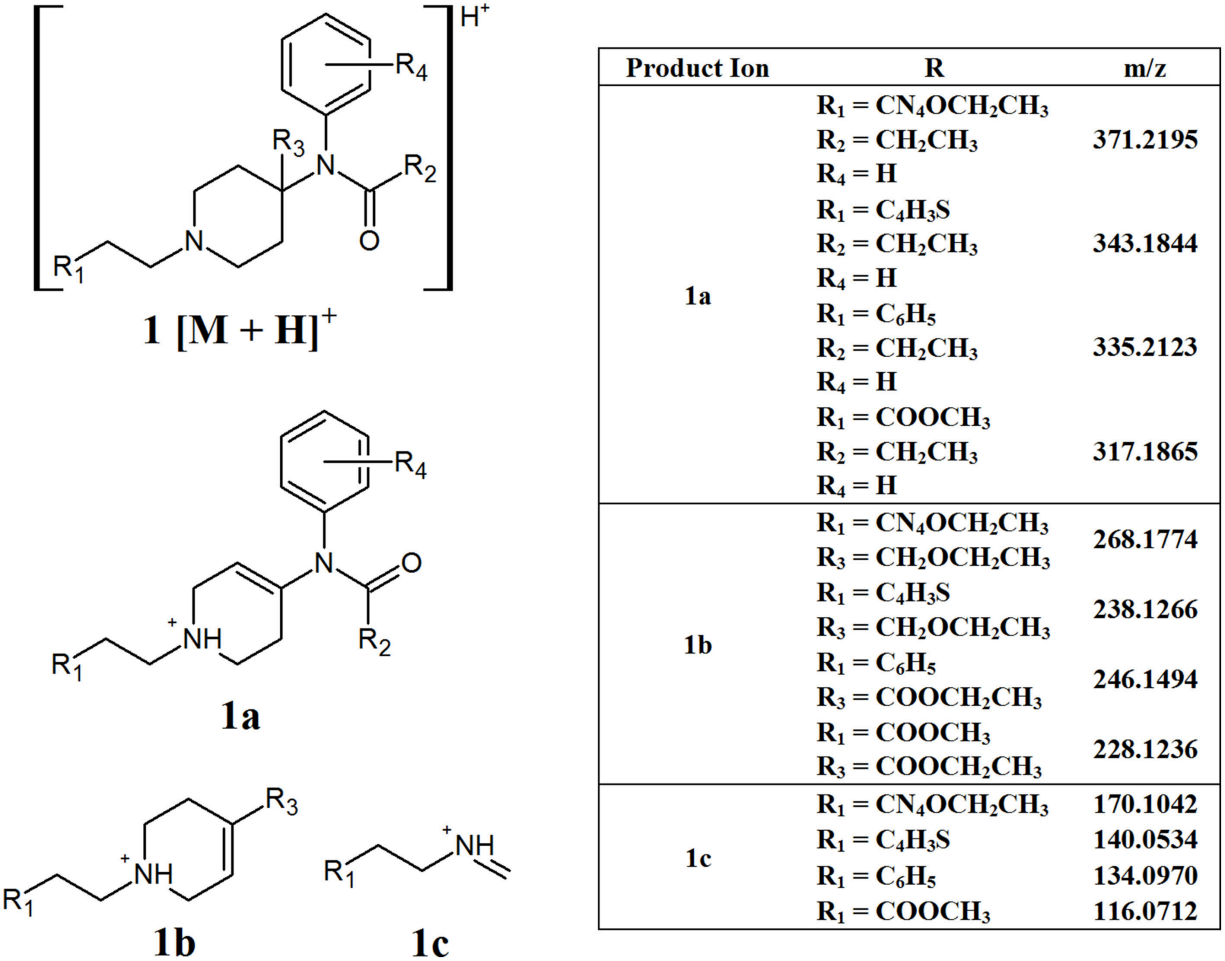
Proposed structures of fragments observed for NPFs containing differing tails and C9 side chains, showing molecular ion **(1)** and common product ions **(1a−1c)**.

The generic structure of fentanyl (Figure 1, structure **A**) shows many different sites that can be altered to produce various analogs. This can lead to different CID pathways being observed depending on the position of substituents. While this can make it difficult to identify common fragments, some overall patterns can be observed for the different sub-groups of NPFs. While a phenylethyl group is the most common substituent found at R_1_, some analogs contain different tails, including ester groups and heterocyclic rings. Additionally, substitution at the C9 position of the piperidine ring (R_3_) can also be found in some NPFs. Figure 2 highlights some common fragment ions that can be found from NPFs containing differing functional groups at these locations.

The main fragments present in the MS^2^ of these compounds may arise from the loss of either the C9 side chain (R_3_) or the phenylamide group, resulting in fragments **1a** and **1b**, respectively. Evidently the exact masses of these resultant product ions would be highly dependent on the various functional groups present in the structure, especially the tail group at R_1_. The other common product ions found in this subclass of compounds, **1c**, may arise from the inter-ring cleavage of the piperidine ring. Similarly to **1a** and **1b**, the exact mass of this fragment will vary depending on the functional group present at R_1_. While the differing masses of the product ions formed is not ideal from a non-targeted screening perspective, understanding the pattern of fragmentation that the molecules undergo is important to help elucidate the structure of possible unknown compounds.

**Figure 3:**
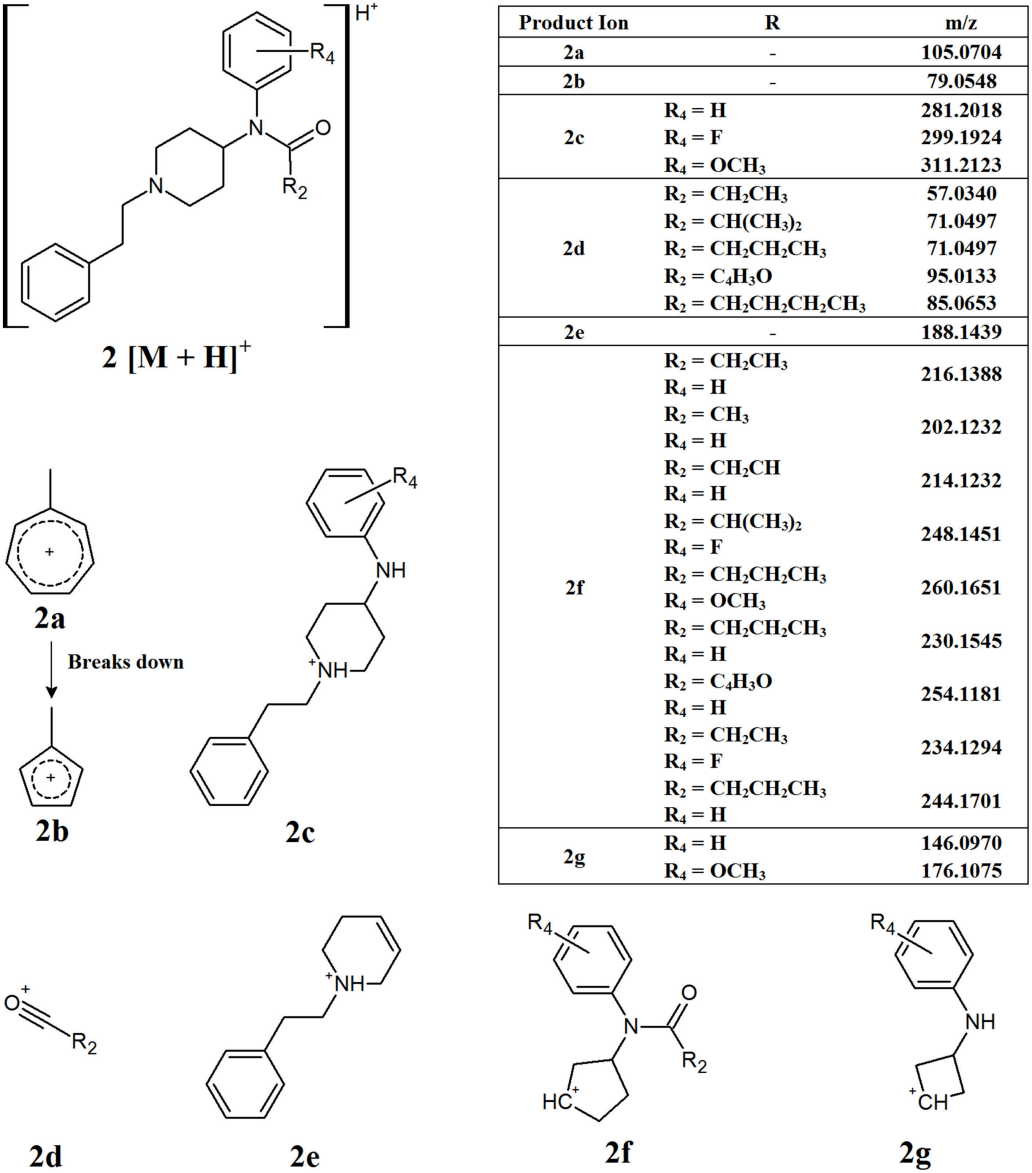
Proposed structures of fragments observed for NPFs containing a phenylethyl tail and lacking a C9 side chain, showing molecular ion **(2)** and common product ions **(2a−2g)**.

As mentioned previously, the most common functional group found at R_1_ is an aromatic ring, causing the compound to have a phenylethyl tail off the piperidine ring. Consequently, the most common points of modification between the different derivatives are the R_2_ and R_4_ groups (Figure 1, structure **A**). Figure 3 presents the common product ions identified when this core structure is present and there is no additional side chain present on the piperidine ring (R_3_). The first common product ion can be found at an m/z of 105.0704 which is representative of a methyl-substituted tropylium ion (**2a**). This fragment is very common for alkyl-substituted benzenes and will often break down to form the methyl-substituted aromatic cyclopentadienyl cation, fragment **2b**, with an m/z of 79.0548 (Pavia et al., 2014). While these product ions are quite common, and therefore not specific to NPFs, their presence in addition to other common fragments can help confirm the presence of these compounds. Product ions **2c** and **2d** may be formed from the cleavage between the nitrogen and the carbonyl group of the amide side chain. The m/z of fragment **2c** may change with substitution of the aromatic ring, however many derivatives have an unsubstituted aromatic ring, leading to a product ion with an m/z of 281.2018. The functional groups at R_2_ on fragment **2d** are much more varied, leading to different m/z values being observed, however the loss between the molecular ion and fragment **2c** can give an indication of the expected m/z for fragment **2d**. The further loss of the nitrogen and aromatic ring from fragment **2c** gives rise to fragment **2e**, with an m/z of 188.1439. While it has not been confirmed whether this product ion is formed directly from the fragmentation of the molecular ion, or if further fragmentation of another product ion has occurred, it is not of great importance for non-targeted screening purposes. The important information in this case is that a product ion with the observed m/z is present in the samples analyzed. The loss of the phenylethyl tail and the collapse of the piperidine ring leads to the formation of fragment **2f**. Once again, the exact m/z of this fragment will vary due to the presence of the amide side chain, which can vary dramatically. Finally, fragment **2g** can form from the collapse of the piperidine ring and cleavage between the nitrogen and carbonyl group of the amide side chain. Similarly to fragment **2c**, fragment **2g** may vary with substitution of the aromatic ring, however it is most commonly found with an m/z of 146.0970 when no substituent is present. Interestingly, the two fluorine-containing derivatives analyzed in this study (4-fluoroisobutyryl fentanyl **A5** and meta-fluoro fentanyl **A9**, Figure 1) both showed fragments at m/z 146.0970. This indicates that fluorine may have been lost before the formation of this fragment, as no corresponding fragment was observed at m/z 165.0954 which would have been expected for the fluorine-substituted product ion.

While all the common product ions identified were not observed for all NPFs, the large number identified meant that a combination of multiple common fragments were present across all derivatives analyzed. The presence of a combination of these common fragments in an unknown sample can provide a strong indication that an NPF may be present within the sample. The core structure displayed by these compounds, especially those related to structure **2**, allows for the use of a smaller number of fragments to identify the presence of a broad range of compounds. More importantly, some of the fragments presented in Figure 3 have little or no variation in their m/z, regardless of the alterations made to core structure. This means that these product ions could potentially be used to screen for previously unknown derivatives, provided that they contain the same core structure. This is of great importance for non-targeted analysis, since the structures of NPFs are unknown.

#### AH Series Opioids

**Figure 4:**
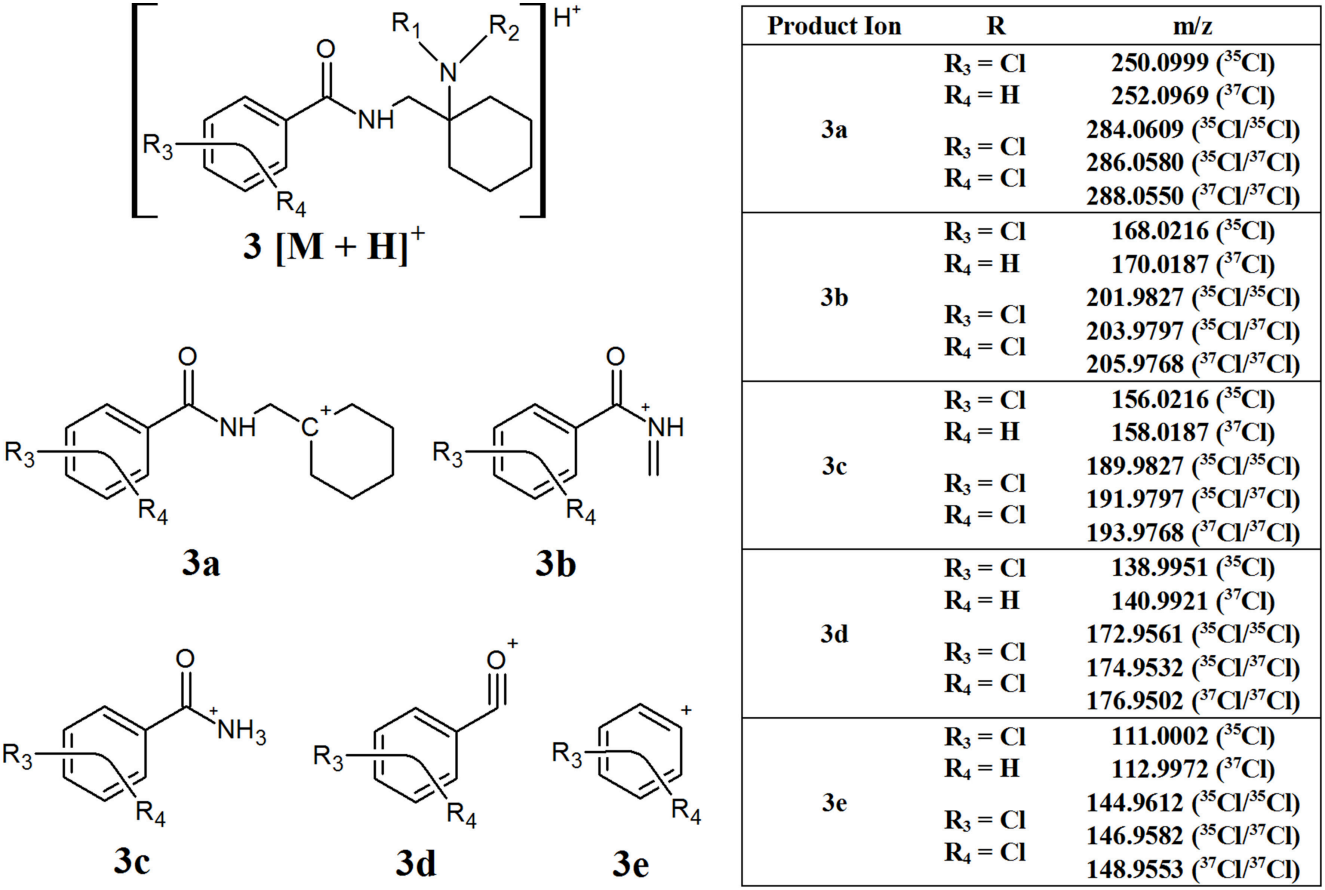
Proposed structures of fragments observed for AH series opioids, showing molecular ion **(3)** and common products ions **(3a−3e)**.

The AH series of synthetic opioids fragment quite predictably, breaking down from the cyclohexane ring toward the aromatic group (Figure 4, structure **3**). The first product ion is generated from the loss of the amine side chain attached to the cyclohexane ring to form **3a**. While the mass loss caused by this fragmentation can vary depending on the structure of the amine side chain, most AH series opioids have one or two Cl substituents on the aromatic group, resulting in product ion **3a** having an m/z of 250.0999 (^35^Cl), 252.0969 (^37^Cl), 284.0609 (^35^Cl/^35^Cl), 286.0580 (^35^Cl/^37^Cl), or 288.0550 (^37^Cl/^37^Cl), respectively. The next major fragments form as a result of the loss of the cyclohexane group and shortening of the amide side chain, giving rise to product ions **3b** and **3c**, respectively. The loss of the amine group and finally the carbonyl group attached to the aromatic ring results in the formation of the final characteristic fragments, **3d** and **3e**, respectively. Fragments **3b**–**3e** may have different m/z values depending on whether they contain one or two Cl substituents, similar to **3a**, however all the compounds within this subclass of synthetic opioids will present a fragment with one of these m/z values. The common m/z produced by these fragments highlight the possibility of using them as targets for screening techniques to detect the presence of this series of NSOs.

#### U Series Opioids

The U series compounds which do not contain a methylene spacer (Figure 1, structure **E**) are constitutional isomers of the compounds within the AH series (Figure 1, structure **B**). This means that, while the structures of the fragments are slightly different, they have identical theoretical m/z values. When it comes to non-targeted analysis, this can be a double-edged sword. On the one hand, having the same m/z values means that a smaller number of common product ions can be used to capture a broader range of compounds. Conversely, the presence of multiple fragment ions with the same m/z reduces the effectiveness of techniques such as class prediction models, making it difficult to determine the identity of these isomers on their fragmentation pattern alone.

**Figure 5:**
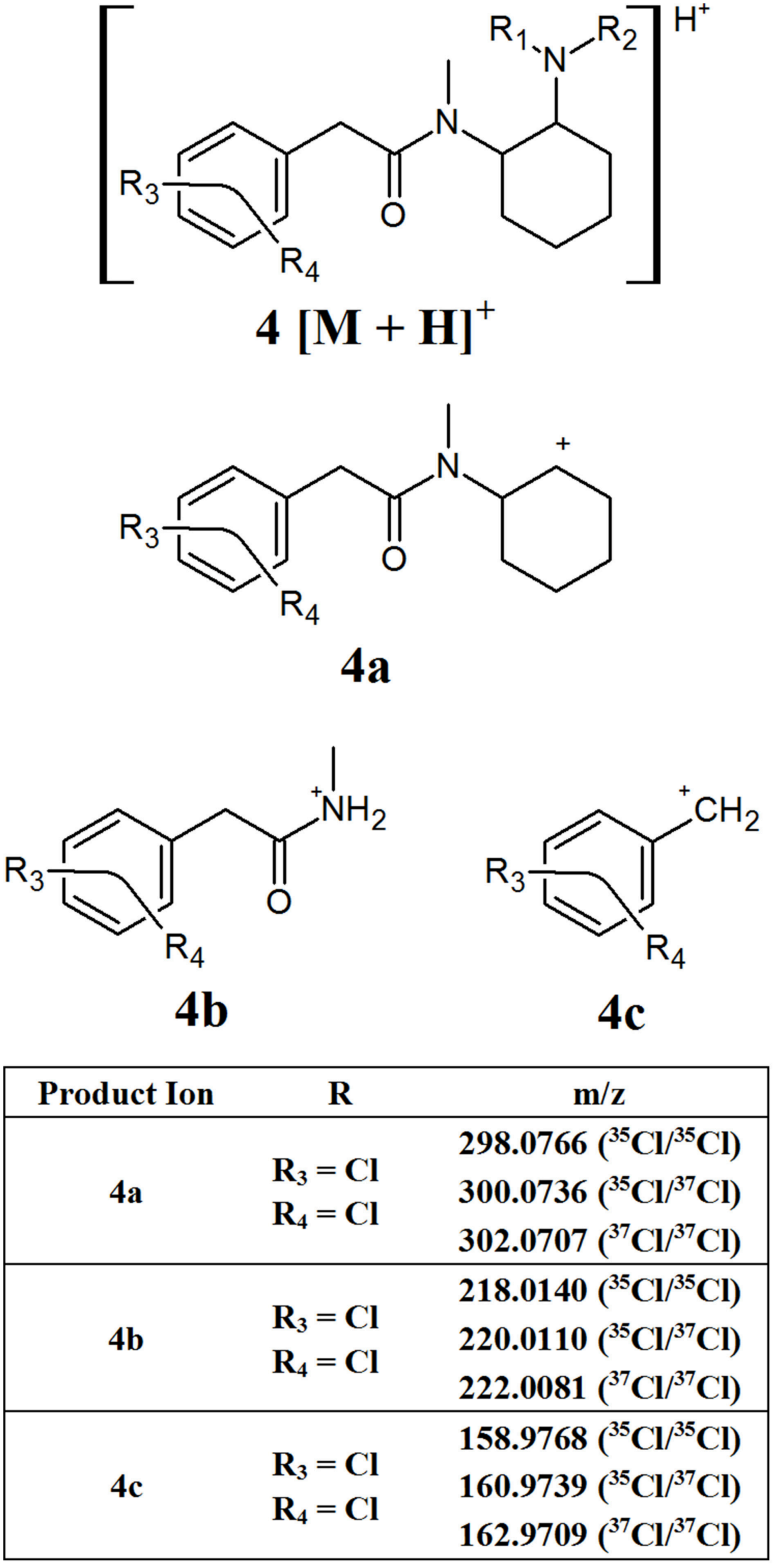
Proposed structures of fragments observed for U series opioids containing a methylene spacer, showing molecular ion **(4)** and common product ions **(4a−4c)**.

The U series compounds which do contain a methylene spacer (Figure 1, structure **D**) fragment in a similar manner, however the presence of the spacer changes the m/z of the product ions observed, allowing them to be distinguished from other NSOs. Figure 5 provides an overview of the major common fragments observed from the CID of these compounds. Similarly to the AH series compounds, the loss of the amine group on the cyclohexane ring may produce fragment **4a**. All the compounds analyzed in this study containing the additional methylene spacer had two Cl substituents on the aromatic ring, leading to fragment **4a** having a theoretical m/z of 298.0766 (^35^Cl/^35^Cl), 300.0736 (^35^Cl/^37^Cl), and 302.0707 (^37^Cl/^37^Cl). Changing the functional groups on the aromatic ring would lead to different m/z values being observed for this fragment, as was seen with the AH series compounds, however the two Cl groups appears to be the most common structure found. The subsequent loss of the cyclohexane ring and the amide group produced fragments **4b** and **4c**, respectively. Once again, these fragments may have different m/z values, dependent on the functional groups present on the aromatic ring, however the fragmentation pattern remains consistent.

#### W Series Opioids

**Figure 6:**
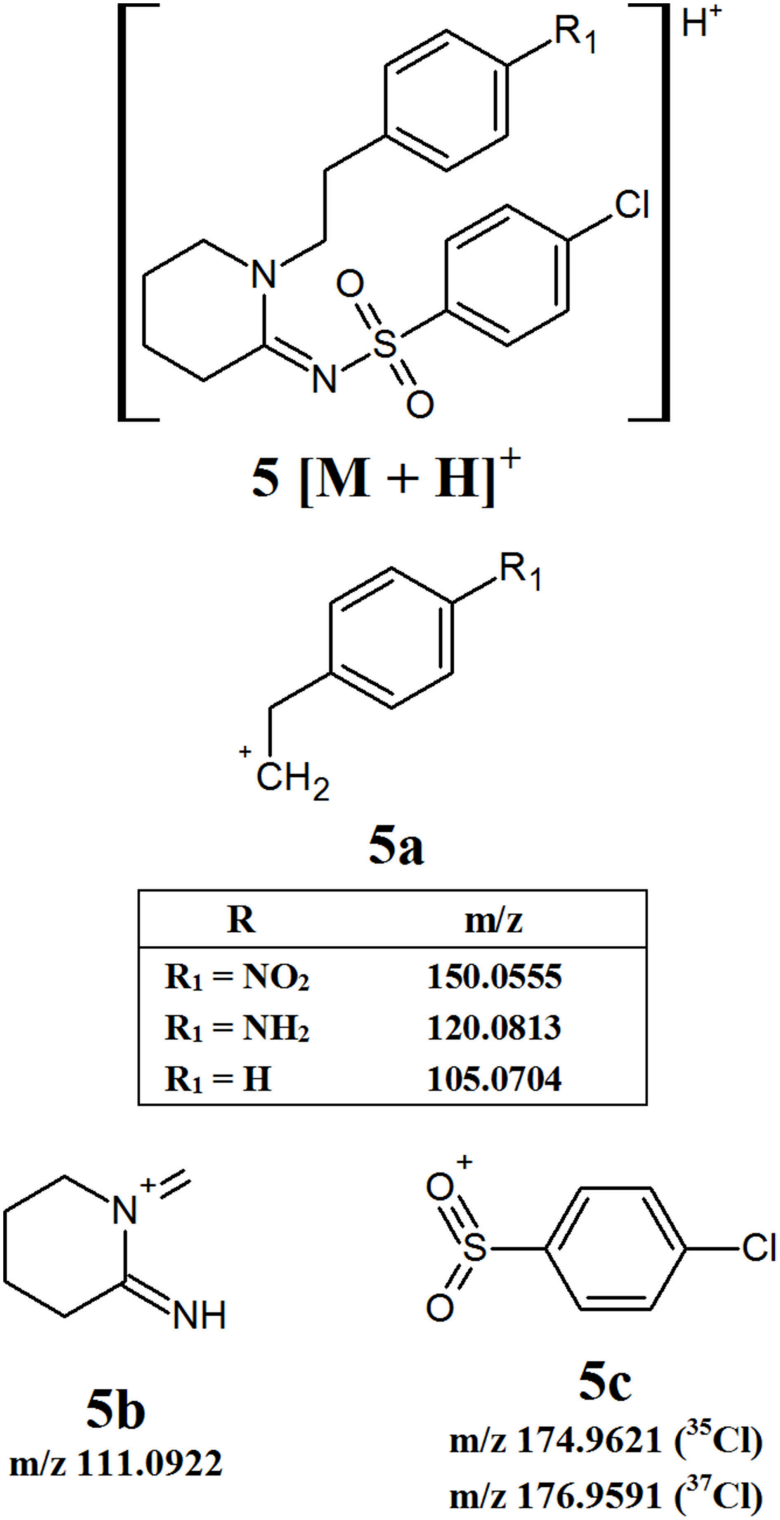
Proposed structures of fragments observed for W series opioids, showing molecular ion **(5)** and common product ions **(5a−5c)**.

The W series of synthetic opioids do not have an extensive fragmentation pattern, with only a few product ions being detected. Figure 6 provides an overview of the common fragment ions proposed. This series of compounds only has one main area of structural variation, meaning that some product ions will have the same m/z regardless of which derivative is analyzed. The first product ion identified, **5a**, comes from the loss of one of the aromatic groups attached to the 6-membered heterocyclic ring and contains the differing functional group. This means that the exact m/z of this fragment may vary depending on the substituent attached to the aromatic ring. The remaining two fragments, **5b** and **5c**, respectively, arise from the isolation of the heterocyclic ring and the breaking of the sulfur-nitrogen bond. Given that these fragments do not have any varying functional groups, fragment **5b** will have a consistent m/z of 111.0922. Fragment **5c** will have theoretical m/z values of 174.9621 (^35^Cl) and 176.9591 (^37^Cl). While there may not be as many common fragment ions within this series, there are still some masses which may prove useful targets for the development of screening methods.

#### MT-45

**Figure 7:**
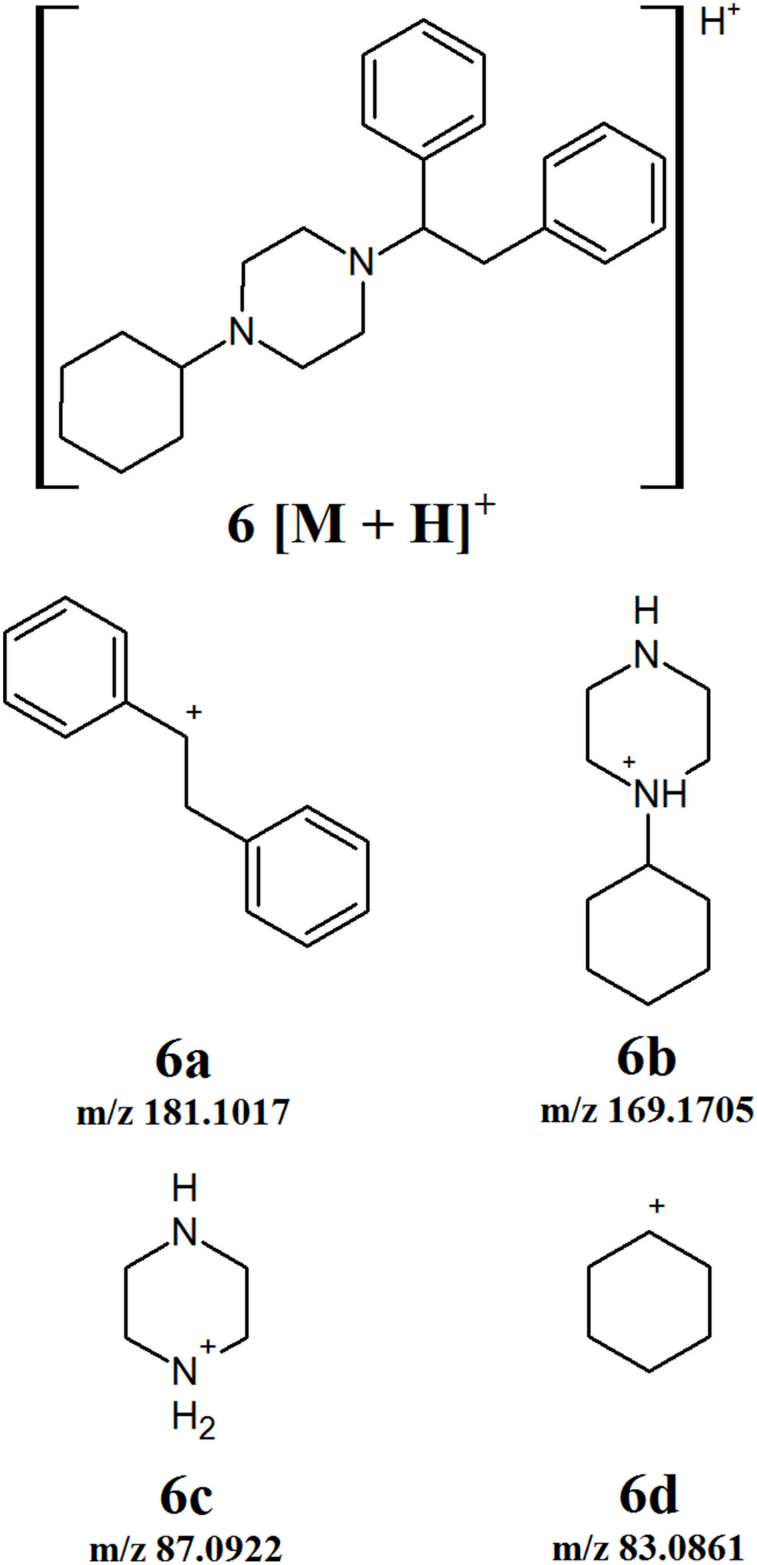
Proposed structures of fragments observed for MT-45, showing molecular ion **(6)** and product ions **(6a−6d)**.

MT-45 is a synthetic opioid that does not fit into any of the other defined classes. The fragmentation of this compound was studied and a number of product ions identified (Figure 7). The first fragmentation involves the cleavage of the bond between the piperazine ring and the dibenzene side chain. This gives rise to product ions **6a** and **6b** which can be observed at m/z 181.1017 and 169.1705, respectively. The subsequent cleavage occurs between the piperazine and cyclohexane rings of **6b**, forming product ions **6c** and **6d** with m/z 87.0922 and 83.0861, respectively. While this fragmentation pattern is for a single compound, there is a possibility for the development of additional derivatives based on this core structure. If such derivatives are encountered, the product ions observed can be compared to those presented in Figure 7 to identify which ones can be considered diagnostic ions for the new subclass.

### Non-targeted Screening

In some ways, the development of new analytical approaches for the detection of NPS is somewhat of an arms race, with forensic toxicologists racing to keep up with the production of novel compounds. From this perspective, methods that take a more holistic approach to the detection of compounds structurally related to known classes of NPS have the distinct advantages over traditional targeted methods. These non-targeted approaches are required for the detection and tentative identification of novel analogs that have not been recorded previously (Pasin et al., 2017a). Samples where these kinds of detections arise can then be flagged for further confirmatory analysis when appropriate CRMs are available. In this context, non-targeted screening refers to the methods used for data acquisition, namely DIA and DDA. When DIA is used, all precursor ions that reach the detector are submitted to CID. On the other hand, DDA involves the selection of a limited number of precursor ions for CID, based on a set of user-defined criteria, which is determined at the start of the experiment (Pasin et al., 2017a). Generally, this data is compared to either in-house databases, which are populated with information obtained from the previous analysis of CRMs, or large online databases for compound identification (Kinyua et al., 2015; Baz-Lomba et al., 2016; Pasin et al., 2017a).

The diagnostic product ions identified in this work for each of the different opioid classes can then be applied to filter through this data to identify possible compounds of interest. A summary of the different diagnostic ions for each opioid class can be found in Table 1. This approach relies on the use of MS/MS (sometimes referred to as MS^2^) data, therefore the effectiveness of these data interpretation methods relies on the selection of an appropriate data acquisition technique (Pasin et al., 2017a). Both DIA and DDA techniques can be used for this purpose, however DIA is less likely to result in the loss of data which might be relevant to the compounds of interest. Since DDA methods have a limited number of precursor ions selected for CID, it is possible that the compound/s of interest may not be subjected to CID, especially when there are a number of abundant endogenous compounds in the matrix being analyzed. While the number of selected precursor ions can be increased, this can be limited by the scan rate and data processing capabilities of the instrument used (Pasin et al., 2017a).

**Table 1:** Summary of diagnostic product ions for each subclass of synthetic opioids.

**Opioid class**	Identifier^*^	**Molecular formula**	**Theoretical m/z**
Fentanyls (phenethyl tail and no C9 side chain)	**2a**	[C_8_H_9_]^+^	105.0704
	**2b**	[C_6_H_7_]^+^	79.0548
	**2e**	[C_13_H_18_N]^+^	188.1439
AH	**3a**	[C_14_H_16_NOR_3_R_4_]^+^	250.0999 (^35^Cl) 252.0969 (^37^Cl) 284.0609 (^35^Cl/^35^Cl) 286.0580 (^35^Cl/^37^Cl) 288.0550 (^37^Cl/^37^Cl)
	**3b**	[C_8_H_6_NOR_3_R_4_]^+^	168.0216 (^35^Cl) 170.0187 (^37^Cl) 201.9827 (^35^Cl/^35^Cl) 203.9797 (^35^Cl/^37^Cl) 205.9768 (^37^Cl/^37^Cl)
	**3c**	[C_7_H_6_NOR_3_R_4_]^+^	156.0216 (^35^Cl) 158.0187 (^37^Cl) 189.9827 (^35^Cl/^35^Cl) 191.9797 (^35^Cl/^37^Cl) 193.9768 (^37^Cl/^37^Cl)
	**3d**	[C_7_H_3_OR_3_R_4_]^+^	138.9951 (^35^Cl) 140.9921 (^37^Cl) 172.9561 (^35^Cl/^35^Cl) 174.9532 (^35^Cl/^37^Cl) 176.9502 (^37^Cl/^37^Cl)
	**3e**	[C_6_H_3_R_3_R_4_]^+^	111.0002 (^35^Cl) 112.9972 (^37^Cl) 144.9612 (^35^Cl/^35^Cl) 146.9582 (^35^Cl/^37^Cl) 148.9553 (^37^Cl/^37^Cl)
U (with methylene spacer)	**4a**	[C_15_H_18_NOR_3_R_4_]^+^	298.0766 (^35^Cl/^35^Cl) 300.0736 (^35^Cl/^37^Cl) 302.0707 (^37^Cl/^37^Cl)
	**4b**	[C_9_H_10_NOR_3_R_4_]^+^	218.0140 (^35^Cl/^35^Cl) 220.0110 (^35^Cl/^37^Cl) 222.0081 (^37^Cl/^37^Cl)
	**4c**	[C_7_H_5_R_3_R_4_]^+^	158.9768 (^35^Cl/^35^Cl) 160.9739 (^35^Cl/^37^Cl) 162.9709 (^37^Cl/^37^Cl)
W	**5b**	[C_6_H_11_N_2_]^+^	111.0922
	**5c**	[C_6_H_4_SO_2_Cl]^+^	174.9621 (^35^Cl) 176.9591 (^37^Cl)

* *Refer to Figures 3–6*.

Using simple data processing methods, namely EICs and neutral loss filtering (NLF), the identified product ions can be used to detect the presence of different synthetic opioids in complex samples. These techniques can be applied manually, however many data processing software packages allow for the automation of these processes. NLF calculates the mass difference between the product ions identified and the precursor ion. The samples can then be interrogated for compounds that contain these specific mass losses. This technique can be a useful adjunct to EICs in situations where the product ions contain variable R groups, meaning that their m/z values will vary, but the neutral molecule which is lost has a consistent mass. For many of the synthetic opioids, this technique has limited value, since many of the neutral losses also contain R groups and, therefore, the exact mass loss will vary. A group where NLF may prove useful are the NPFs that contain side chains at the carbon-9 position of the piperidine ring (Figure 2). All of the common product ions identified for these compounds have R groups present, however, the proposed structure for fragment **1a** arises from the loss of the C9 side chain. In all the compounds analyzed from this group, the side chain consisted of either an ether or ester group with masses of 59.0497 or 73.0290 Da, respectively. In this case, it may be useful to target these two neutral losses to indicate the presence of one of these compounds.

It is important to note, however, that NLF can be more easily applied to data collected using DDA methods, rather than DIA. As reported by Oberacher and Arnhard (2016), the use of DIA leads to the production of chimeric spectra where the software cannot associate product ions with their correct precursor ions and, therefore, neutral losses cannot be calculated. While deconvolution algorithms may be available to help sift through the data, DDA data does not require this extra processing step and, therefore, may benefit more from the use of NLF. These chimeric spectra do not limit the use of diagnostic product ions, however, since all the product ions created will still be present in the spectra. Since some product ions with consistent m/z values have been identified for the different groups, with the exception of the NPFs discussed previously, the use of EICs to target these diagnostic product ions may be the most effective way to detect their presence in unknown samples.

**Figure 8:**
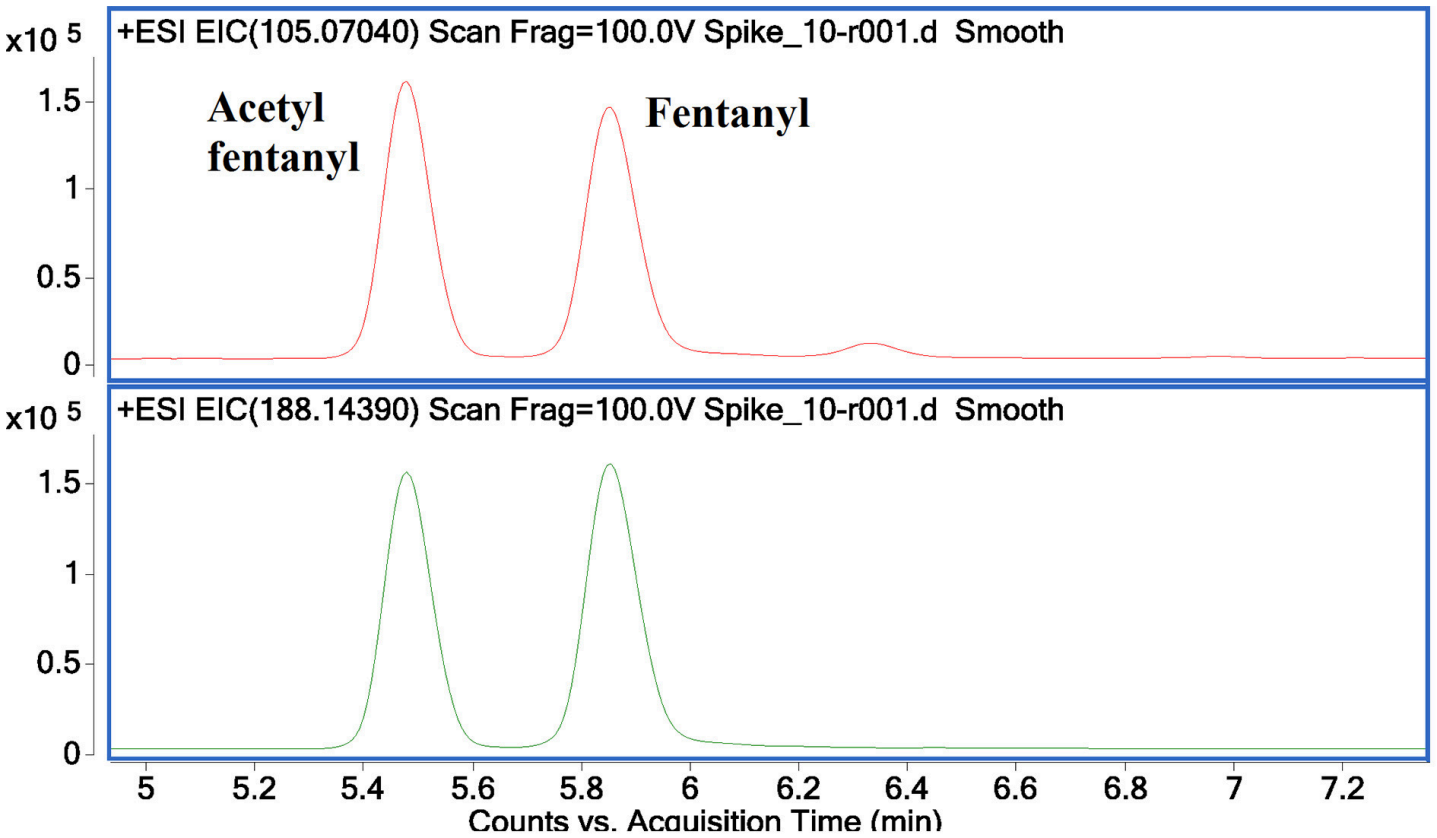
Extracted Ion Chromatograms for diagnostic product ions 105.0704 **(top)** and 188.1439 **(bottom)** showing the identification of acetyl fentanyl and fentanyl in a mixed sample.

Figure 8 shows the EICs for the diagnostic product ions identified with m/z values of 105.0704 and 188.1439. These product ions were found to be common to NPFs containing a phenethyl tail and lacking any substitution at the C9 position of the piperidine ring (Figure 3). It can be seen from these EICs that there are two peaks at 5.5 and 5.85 min which indicate the presence of acetyl fentanyl **A2** and fentanyl **A1** (Figure 1), respectively. For the purpose of this proof-of-concept sample, the MS/MS spectra of the identified peaks were observed and the retention times were compared to a targeted reporting method to confirm the identities of the compounds detected. As mentioned previously, a fragment with an m/z value of 105.0704 is quite common for alkyl-substituted benzenes, therefore, only identifying a peak in this EIC may not be specific to NPFs and requires further confirmation by the presence of other diagnostic product ions or interrogation of the overall MS/MS spectra.

**Figure 9:**
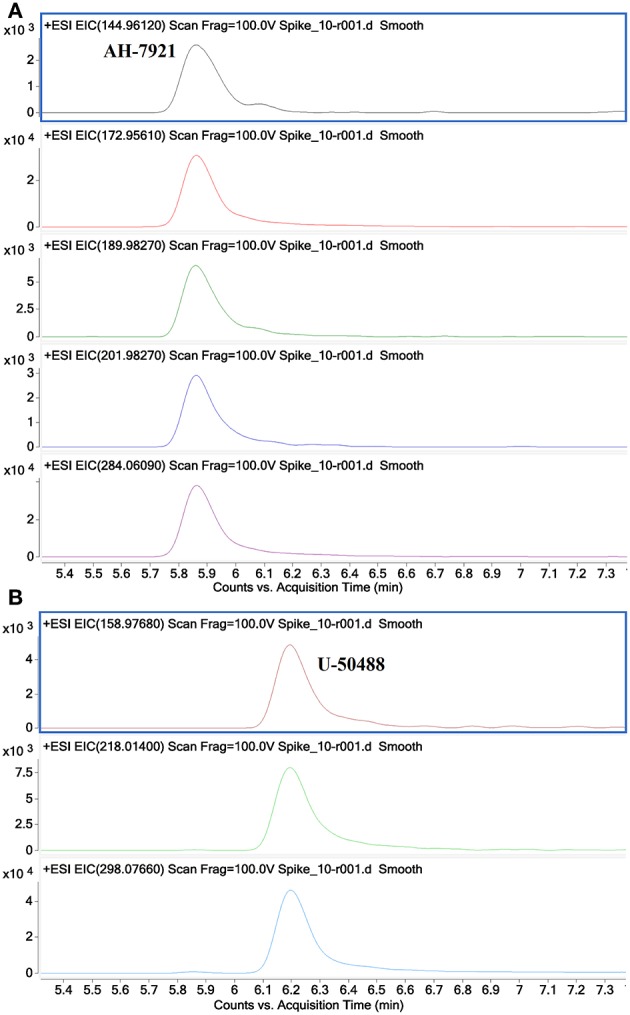
Extracted Ion Chromatograms for diagnostic product ions identified for the AH series opioids 144.9612, 172.9561, 189.9827, 201.9827, 284.0609 **(A)** and for the U series opioids 158.9768, 218.0140 and 298.0766 **(B)** showing the identification of AH-7921 and *U*-50488 in a mixed sample.

Figure 9 shows additional EICs targeting the diagnostic product ions for the AH opioids (**3a−3e**) and U series opioids (**4a−4c**). For this sample, only the m/z values for fragments containing 2 Cl substituents were used. It can be seen that these EICs have indicated the presence of AH-7921 and U-50488 in the sample, with retention times of 5.85 and 6.2 min, respectively. While there are multiple possible m/z values for these fragments due to the different isotopes of Cl which may be present, the detection of any one of these masses may indicate the presence of one of these compounds in the sample being analyzed.

The concentration range of the spikes was evaluated in order to determine an estimated limit of detection (LOD) for this technique in a relevant biological matrix. It was found that, down to a concentration of 0.05 ng/mL, the identified diagnostic fragments could be clearly seen in the spiked samples. At lower concentrations, some of the product ions no longer presented peaks in their EICs, indicating that their abundance was too low to accurately detect. While there were still some product ions that could be detected at lower concentrations, the confidence in the detection of a synthetic opioid would be reduced when fewer diagnostic product ions are identified in a given sample. In the case of fentanyl derivatives containing a phenylethyl tail (Figure 3), if the product ion at *m/z* 188.1439 (Figure 8, bottom) is not detected but the fragment at *m/z* 105.0704 can still be seen, it may cast doubts as to the identity of the detected compound. As previously stated, this fragment is common for any alkyl-substituted aromatic compounds, therefore the specificity of identifying this fragment alone would be limited.

The use of EICs to target common product ions for the different groups of synthetic opioids can be useful for the development of a non-targeted screening method. This method does not rely on extensive MS databases or CRMs to indicate the presence of these compounds. It should be noted that this technique cannot be relied on in isolation and should rather form part of a non-targeted screening workflow. The detection of peaks related to the identified diagnostic product ions can flag samples of interest, which may contain forensically relevant compounds. The MS/MS spectra of the detected peaks can be observed to identify the molecular ion of interest and further, more targeted, analysis can be conducted if necessary. The incorporation of techniques such as this into non-targeted screening workflows can save forensic laboratories time and resources by quickly flagging or eliminating samples and reducing the use of superfluous techniques. This method was applied to 157 race-day equine plasma samples taken during the 2019 Autumn Carnival. While these samples have all shown to be negative for synthetic opioids, it demonstrates the ability of this technique to be incorporated alongside routine analysis.

## Conclusion

Various synthetic opioids, encompassing a number of different subclasses, were subjected to CID using different CEs. A number of different diagnostic product ions were identified for each of the classes analyzed. These diagnostic product ions can be incorporated into non-targeted screening strategies to detect and tentatively classify these compounds without relying of comparison to databases or CRMs.

## Author Contributions

All authors contributed to the conception of the study and assisted with the experimental design. JK prepared and analyzed the samples, proposed the structures of the identified fragments and drafted the manuscript. AC provided opioid samples, blank equine plasma and helped devise the spiking study. RS assisted with the determination of fragment structures. SF supervised the study and assisted with data interpretation. All authors contributed to manuscript preparation, read and approved the submitted version.

### Conflict of Interest Statement

The authors declare that the research was conducted in the absence of any commercial or financial relationships that could be construed as a potential conflict of interest.
