# Supplementary material for: Collision-Induced Dissociation Studies of Synthetic Opioids for Non-targeted Analysis
Source: Front Chem. 2019 May 14;7:331. doi: 10.3389/fchem.2019.00331 (PMC6527801; doi:10.3389/fchem.2019.00331)
Supplement: Supplementary file 1 [file Data_sheet_1.PDF]

Collision-induced Dissociation Studies of Synthetic Opioids for Non-targeted Analysis

Supplementary Material

1 Structures of Compounds Analyzed

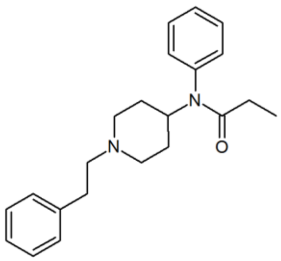

A1

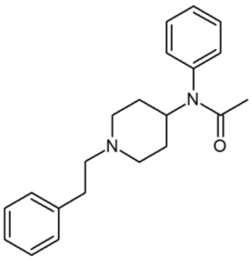

A2

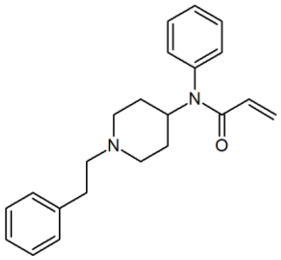

A3

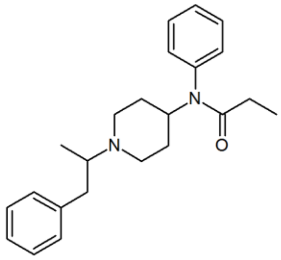

A4

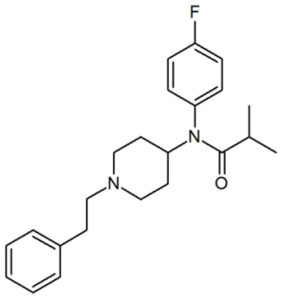

A5

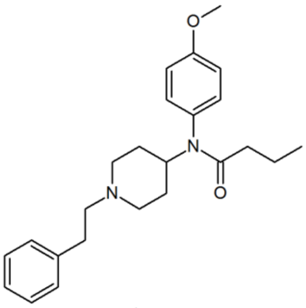

A6

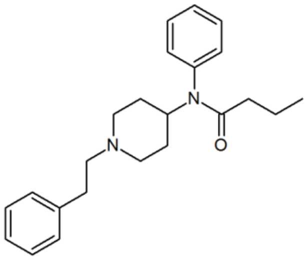

A7

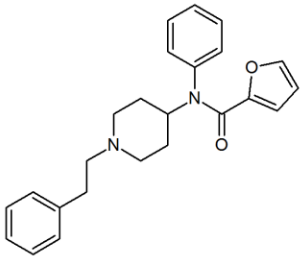

A8

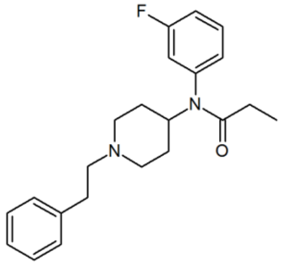

A9

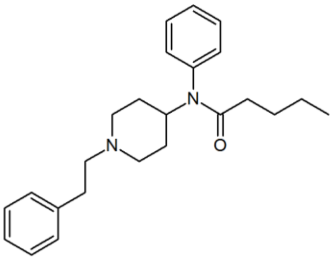

A10

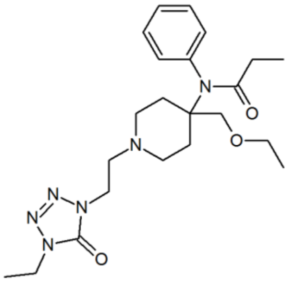

A11

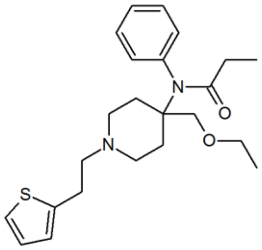

A12

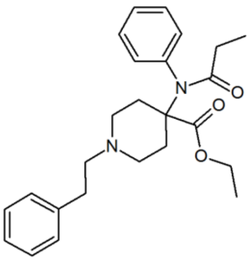

A13

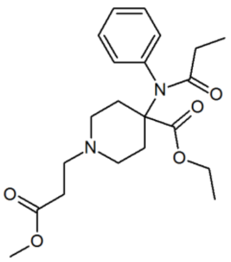

A14

Figure S1. Structures of all fentanyl derivatives analyzed in this study.

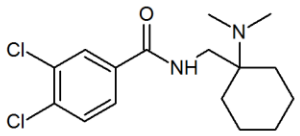

B1

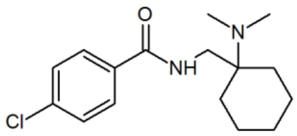

B2

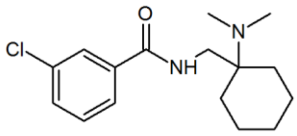

B3

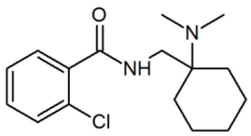

B4

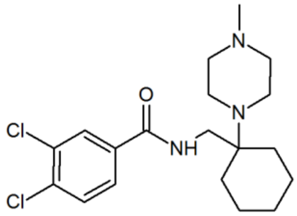

B5

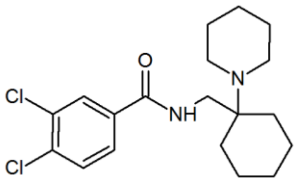

B6

Figure S2. Structures of all AH series opioids analyzed in this study.

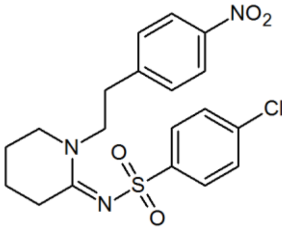

C1

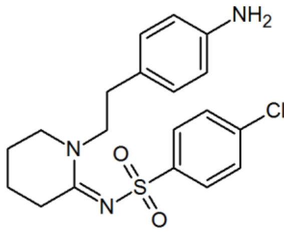

C2

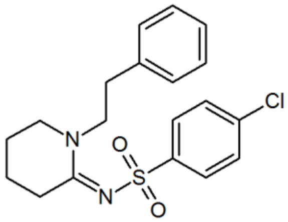

C3

Figure S3. Structures of all W series opioids analyzed in this study.

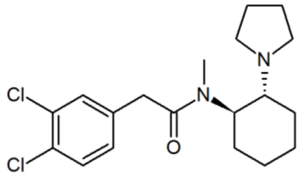

D1

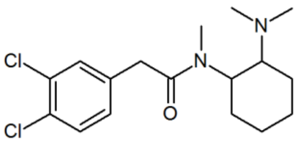

D2

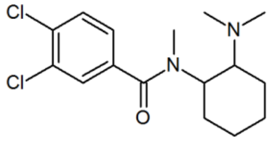

E1

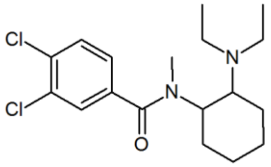

E2

Figure S4. Structures of all U series opioids with and without methylene spacers analyzed in this study.

2 MS/MS Spectra for Analyzed Compounds

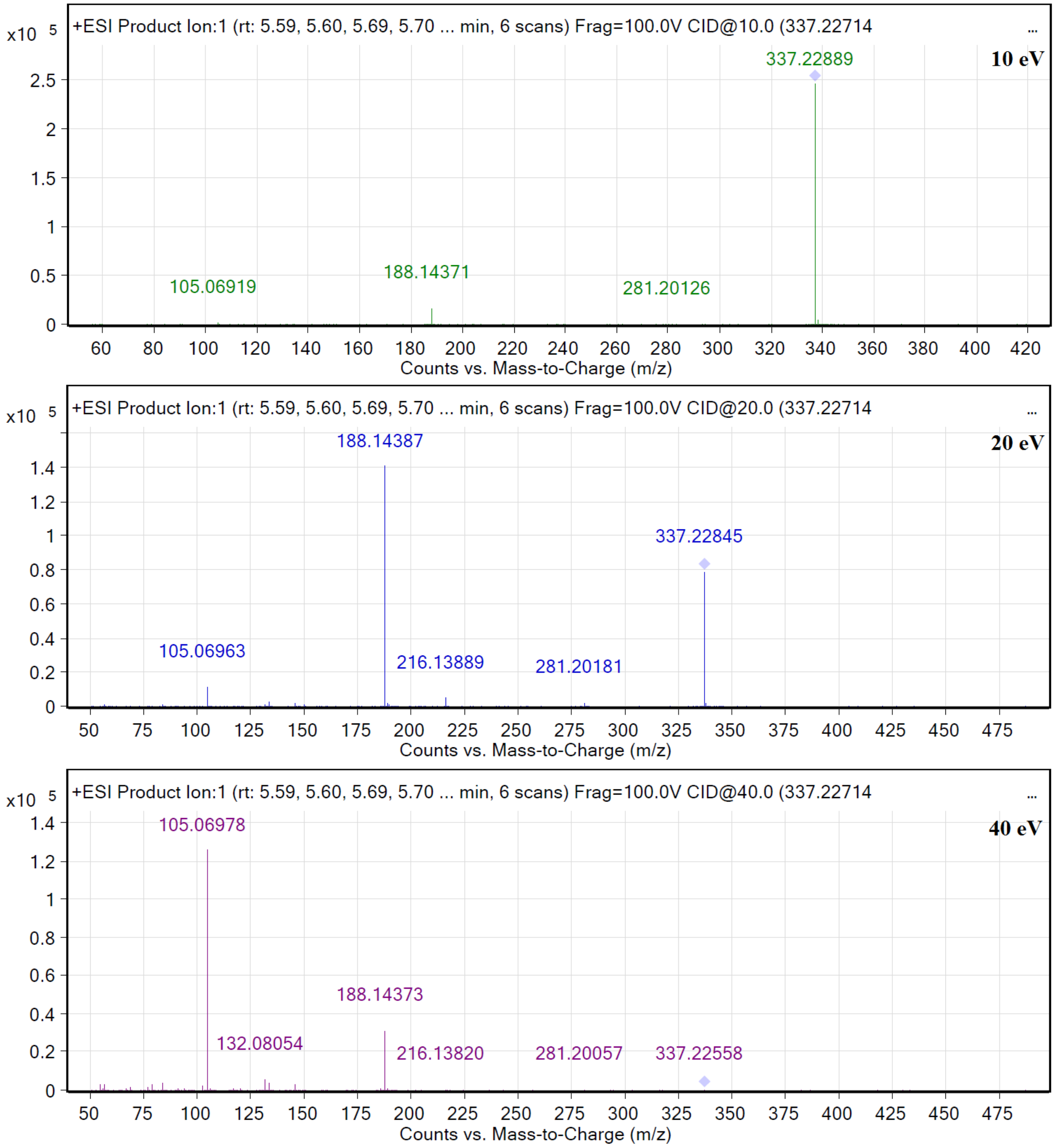

Figure S5. MS/MS spectra obtained from Fentanyl

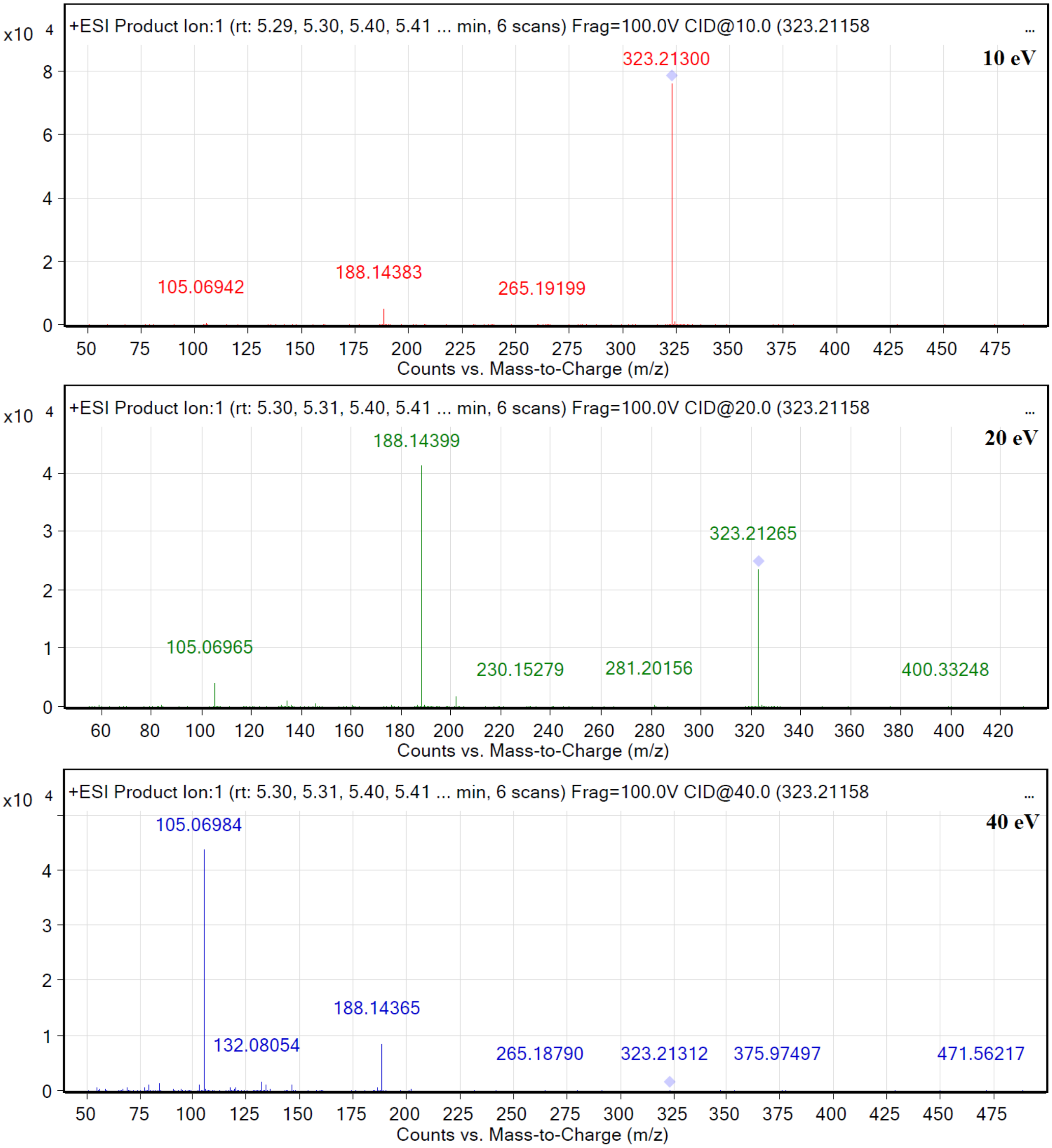

Figure S6. MS/MS spectra obtained from Acetyl fentanyl

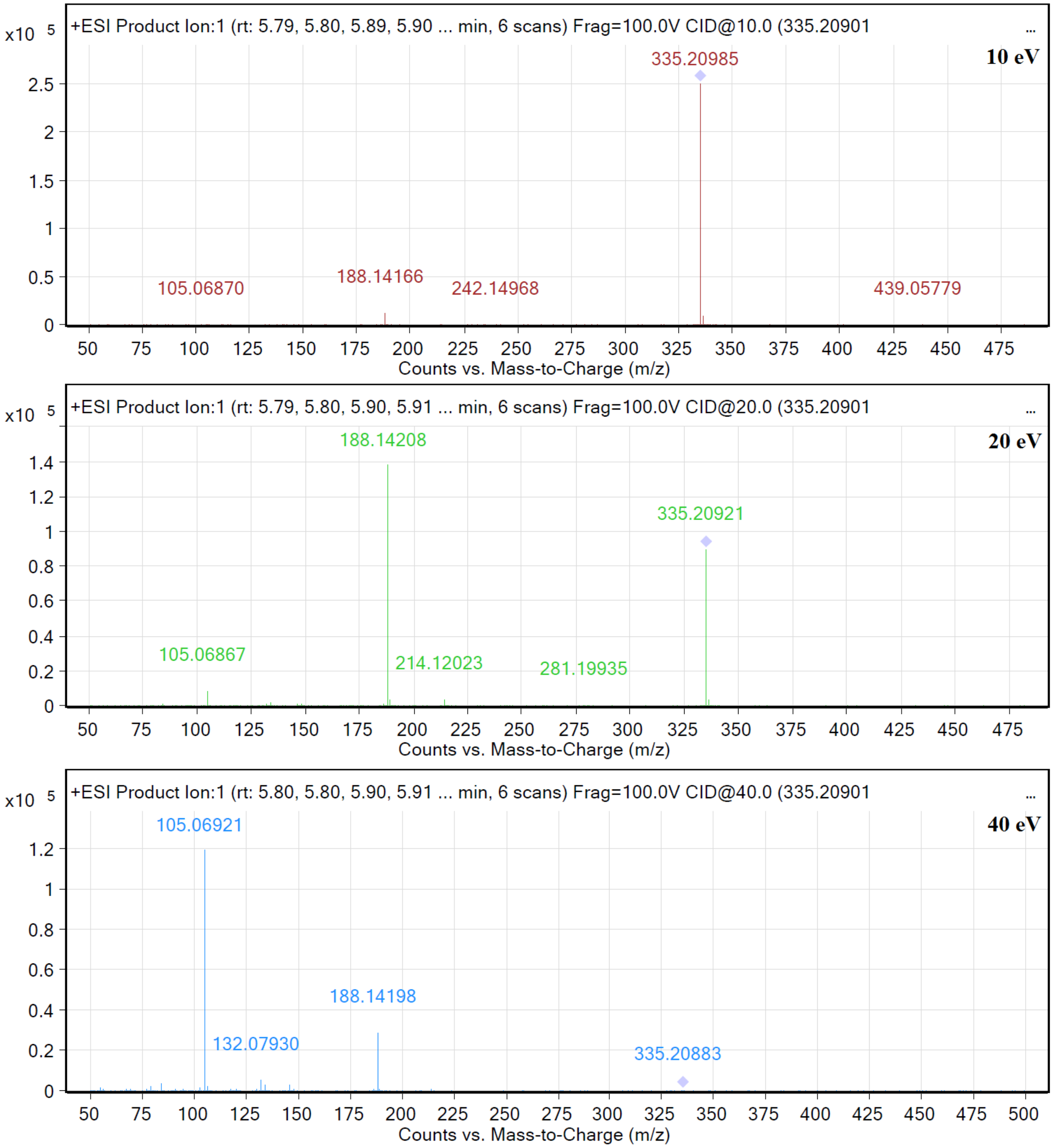

Figure S7. MS/MS spectra obtained from Acryl fentanyl

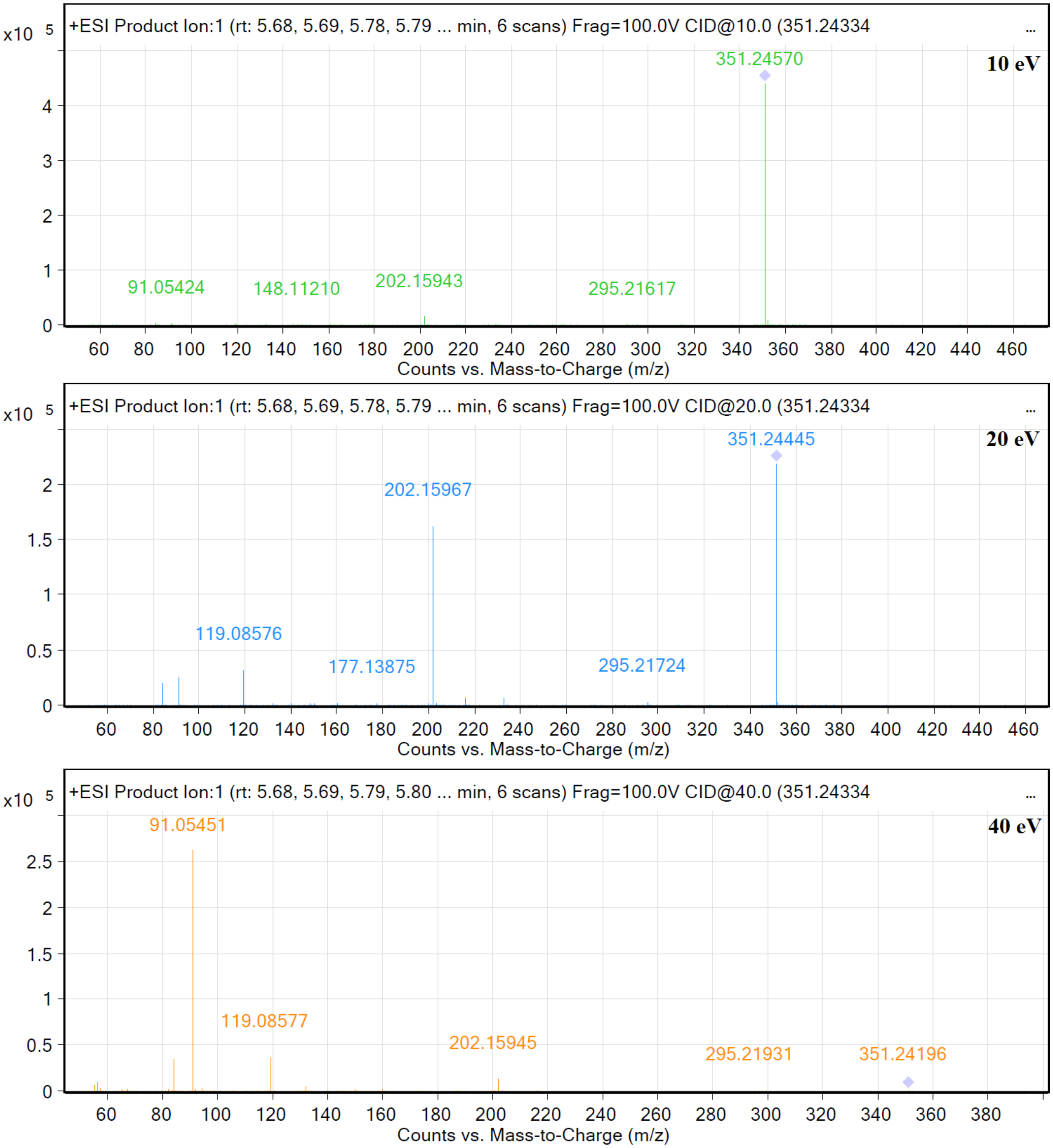

Figure S8. MS/MS spectra obtained from α-methyl fentanyl

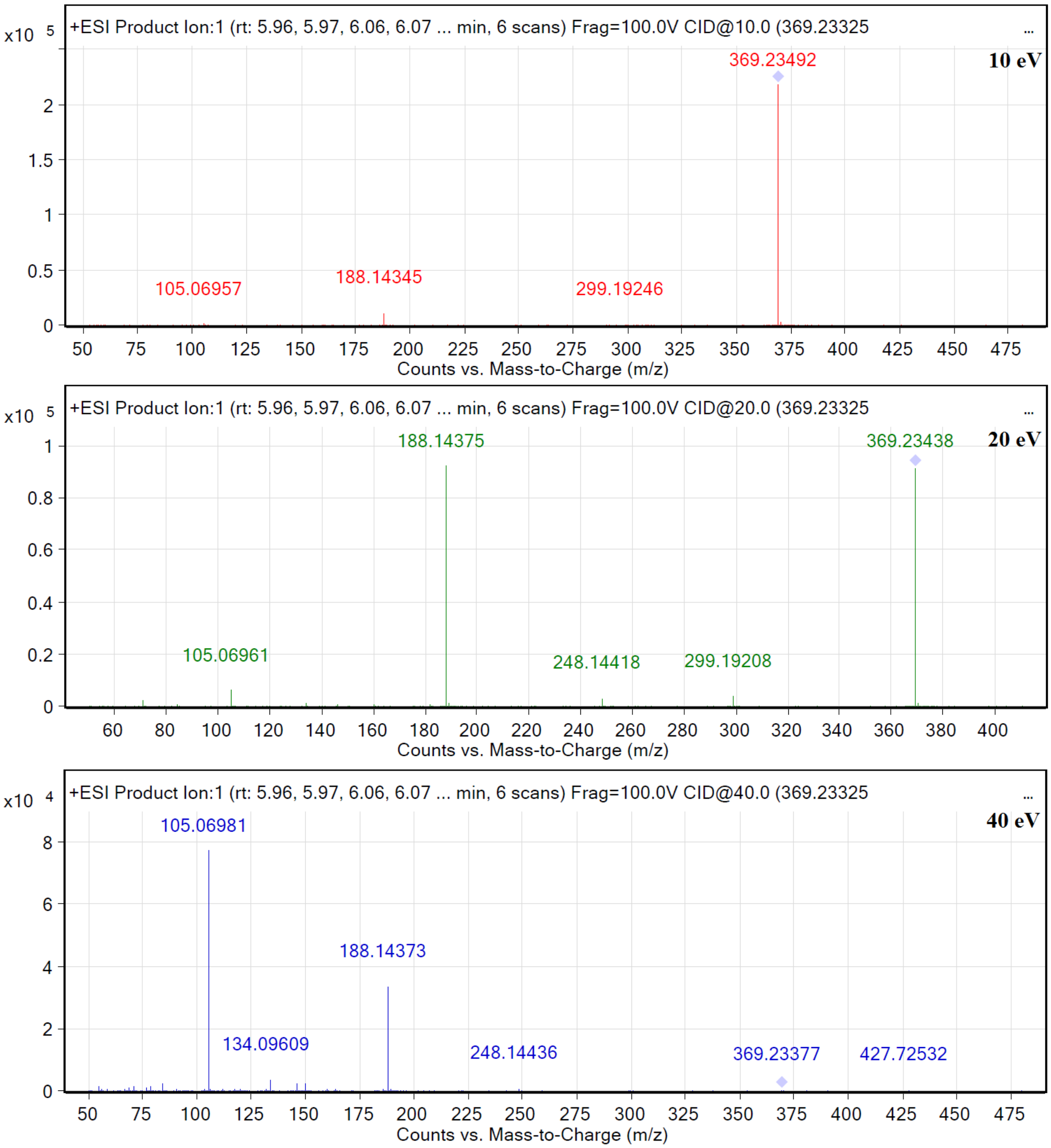

Figure S9. MS/MS spectra obtained from 4-fluoroisobutyrylfentanyl

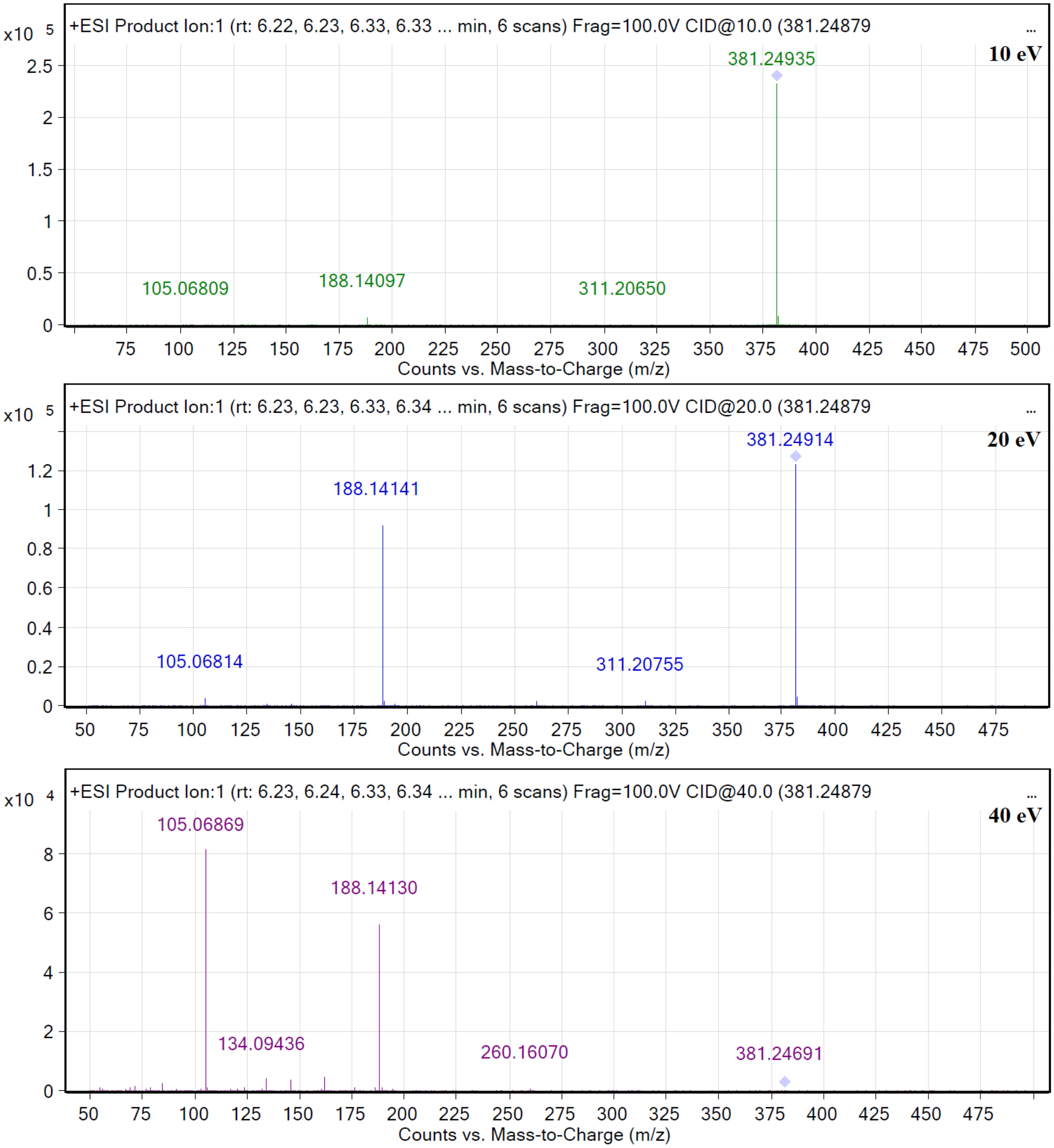

Figure S10. MS/MS spectra obtained from 4-methoxybutyrylfentanyl

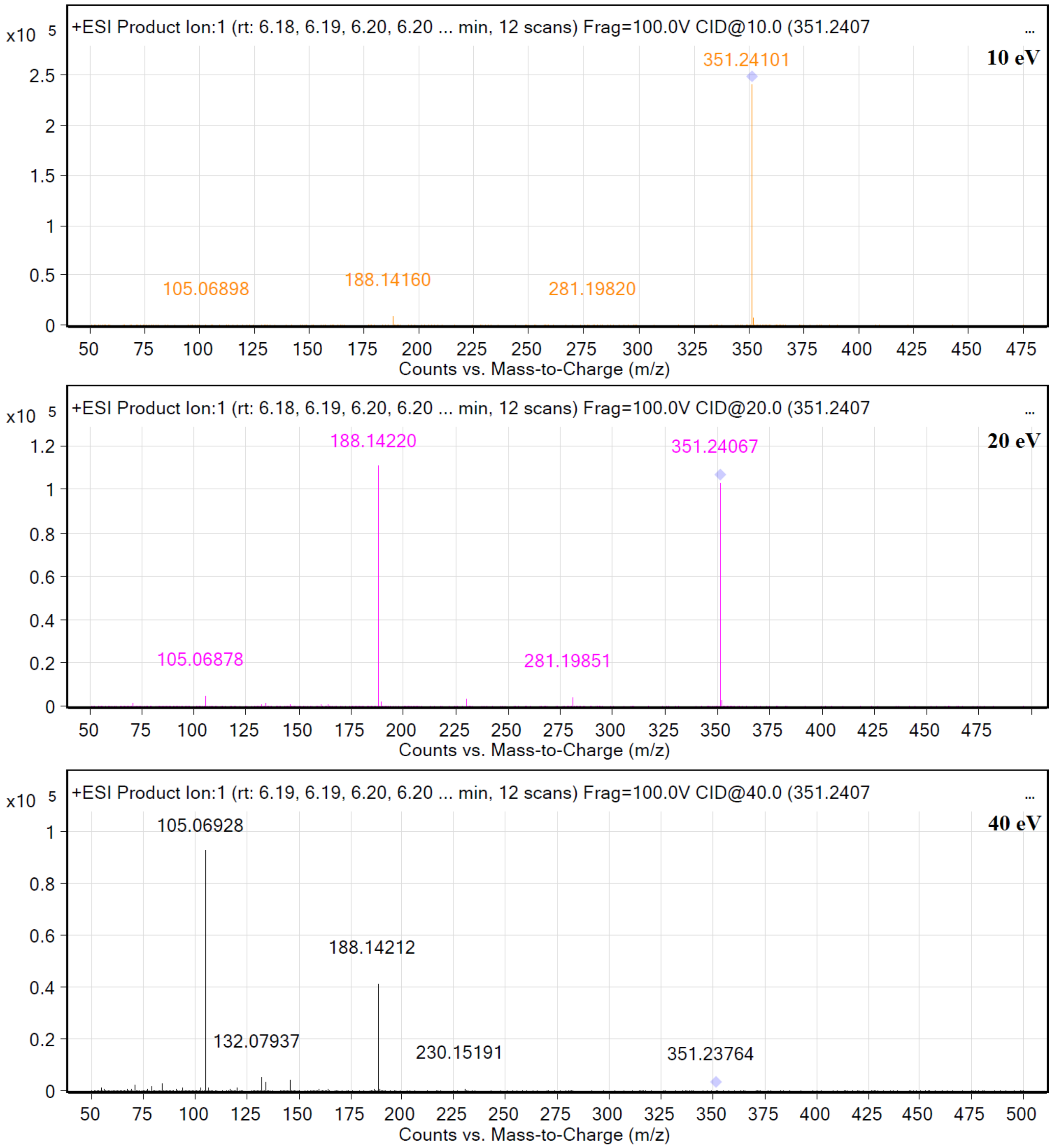

Figure S11. MS/MS spectra obtained from Butyryl fentanyl

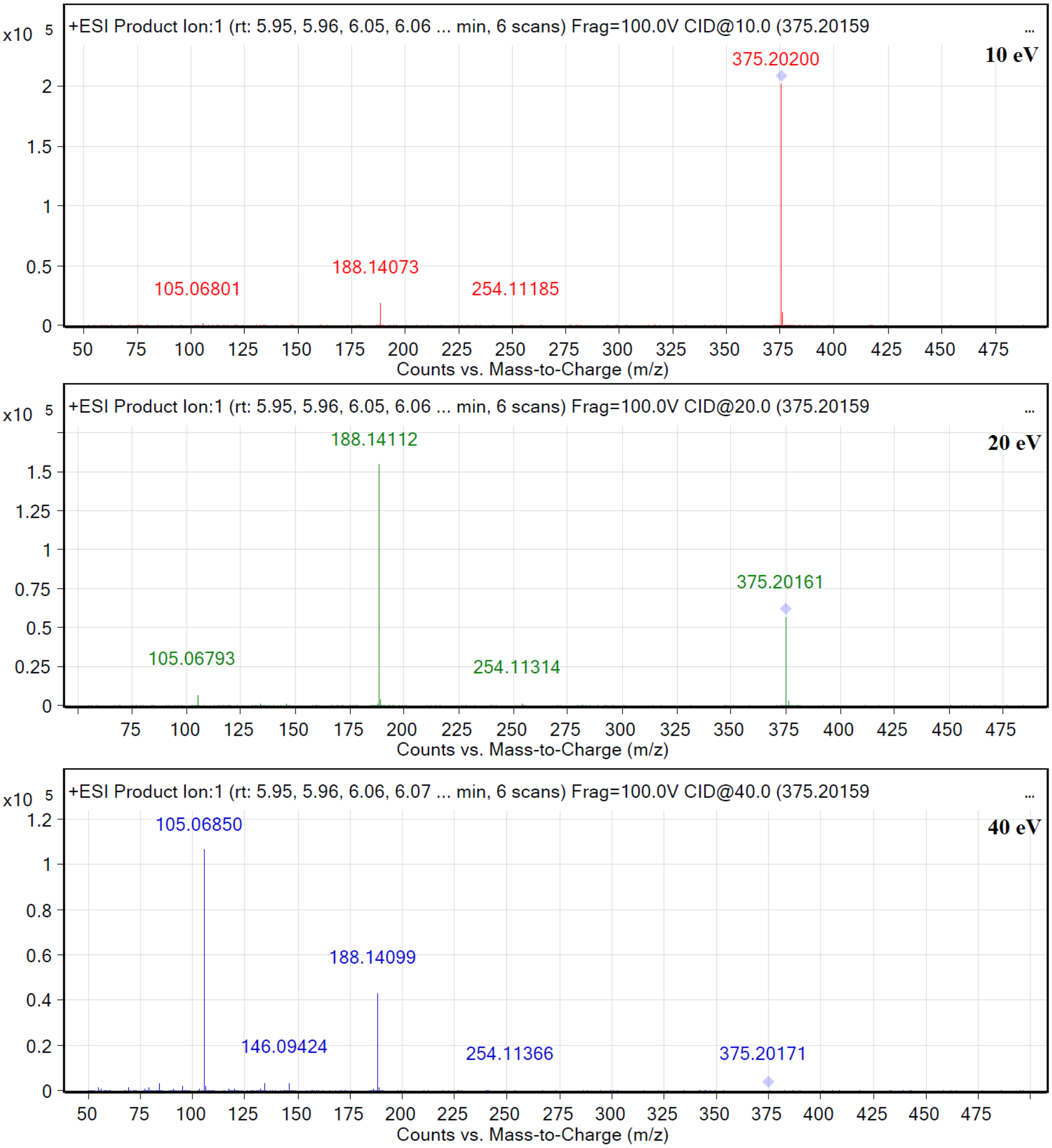

Figure S12. MS/MS spectra obtained from Furanyl fentanyl

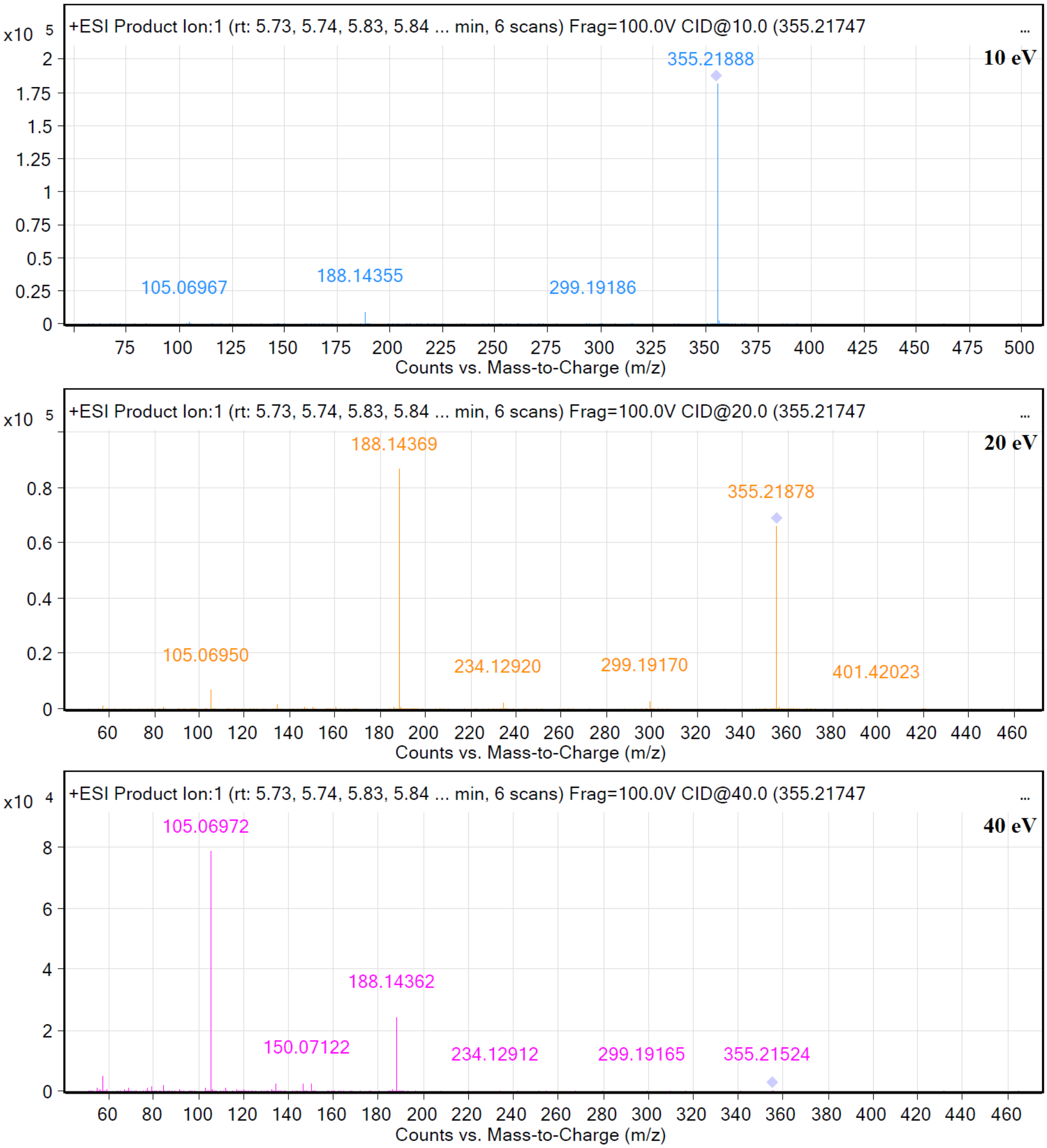

Figure S13. MS/MS spectra obtained from meta-fluoro fentanyl

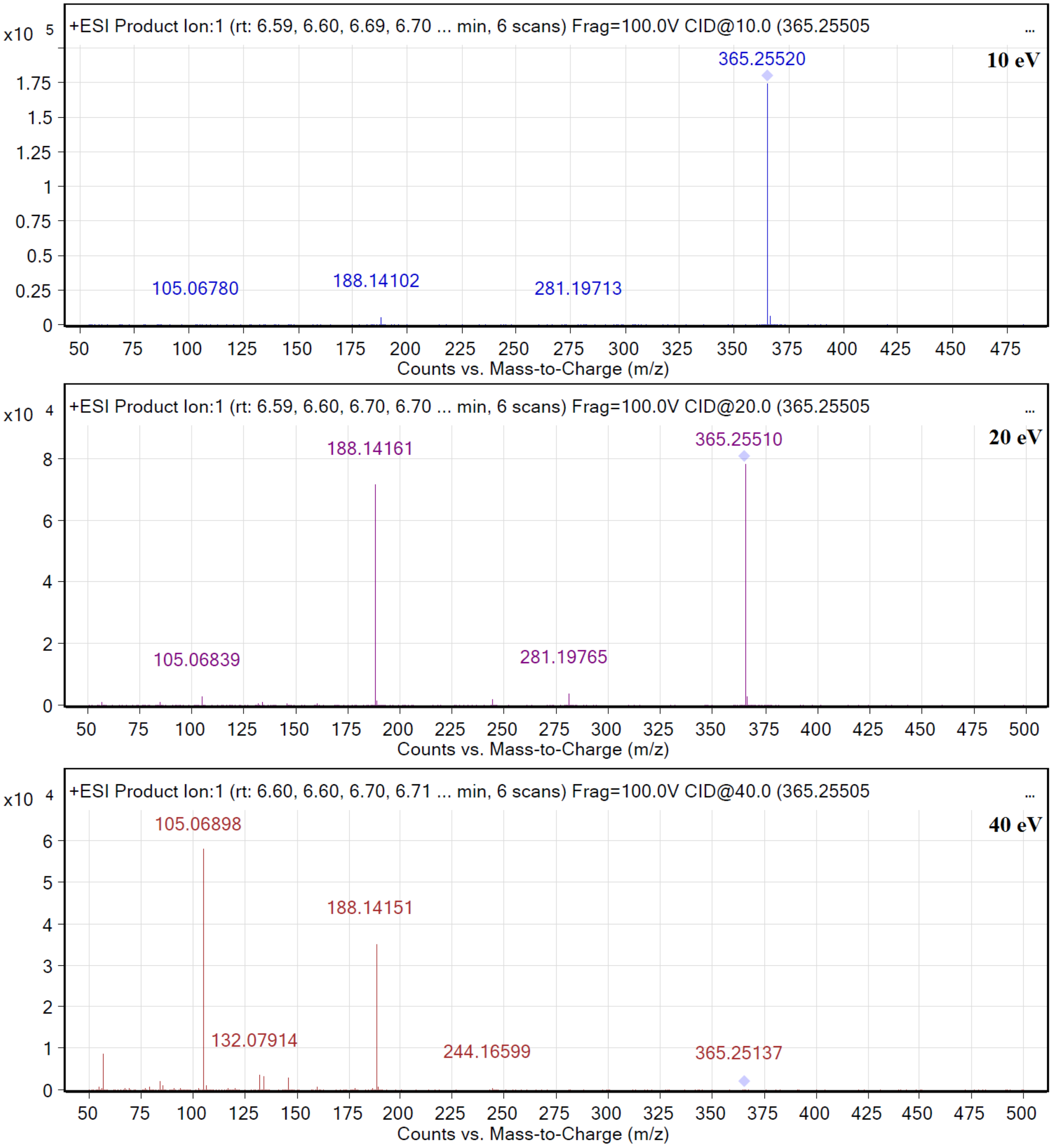

Figure S14. MS/MS spectra obtained from Valeryl fentanyl

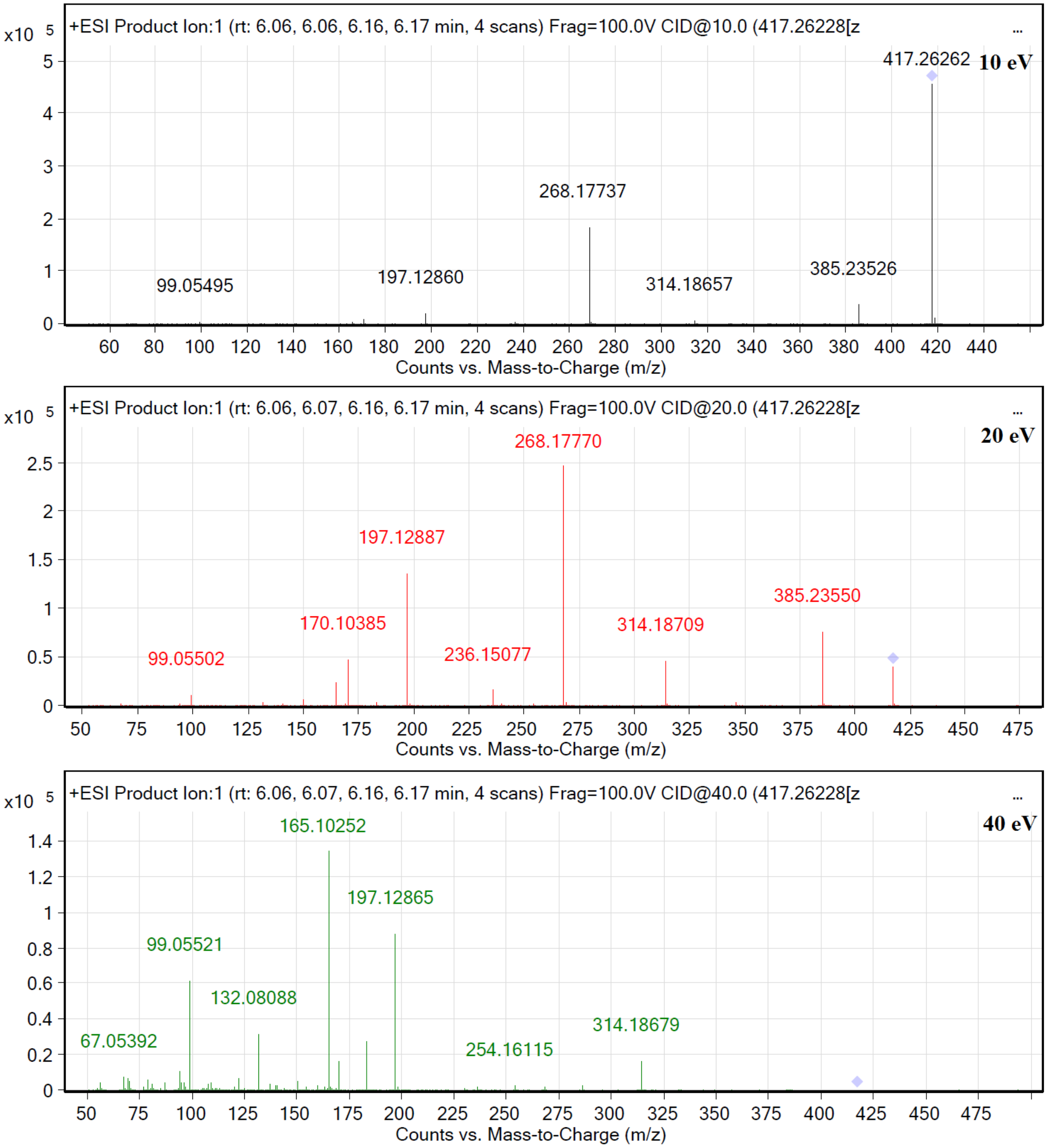

Figure S15. MS/MS spectra obtained from Alfentanil

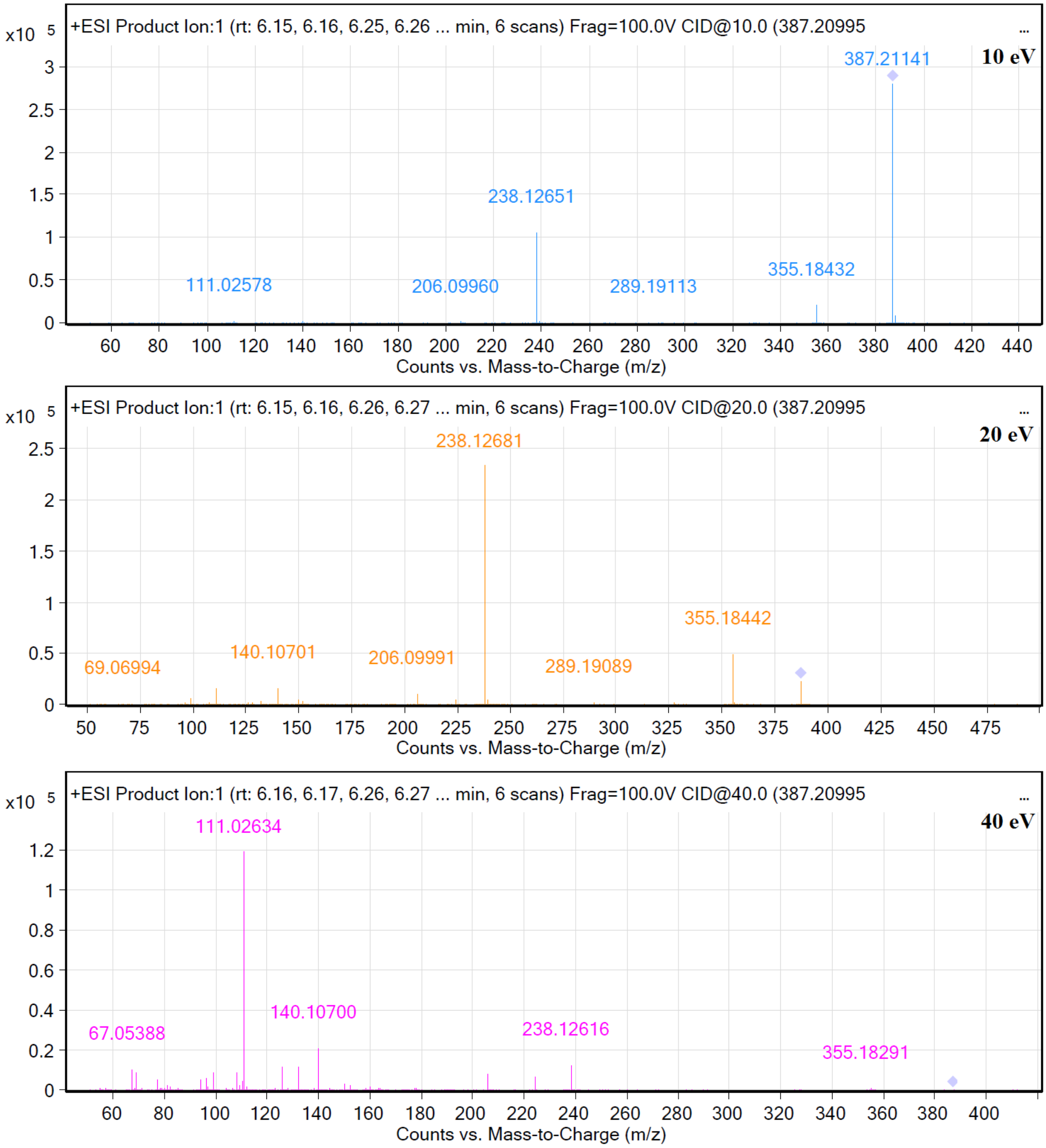

Figure S16. MS/MS spectra obtained from Sufentanil

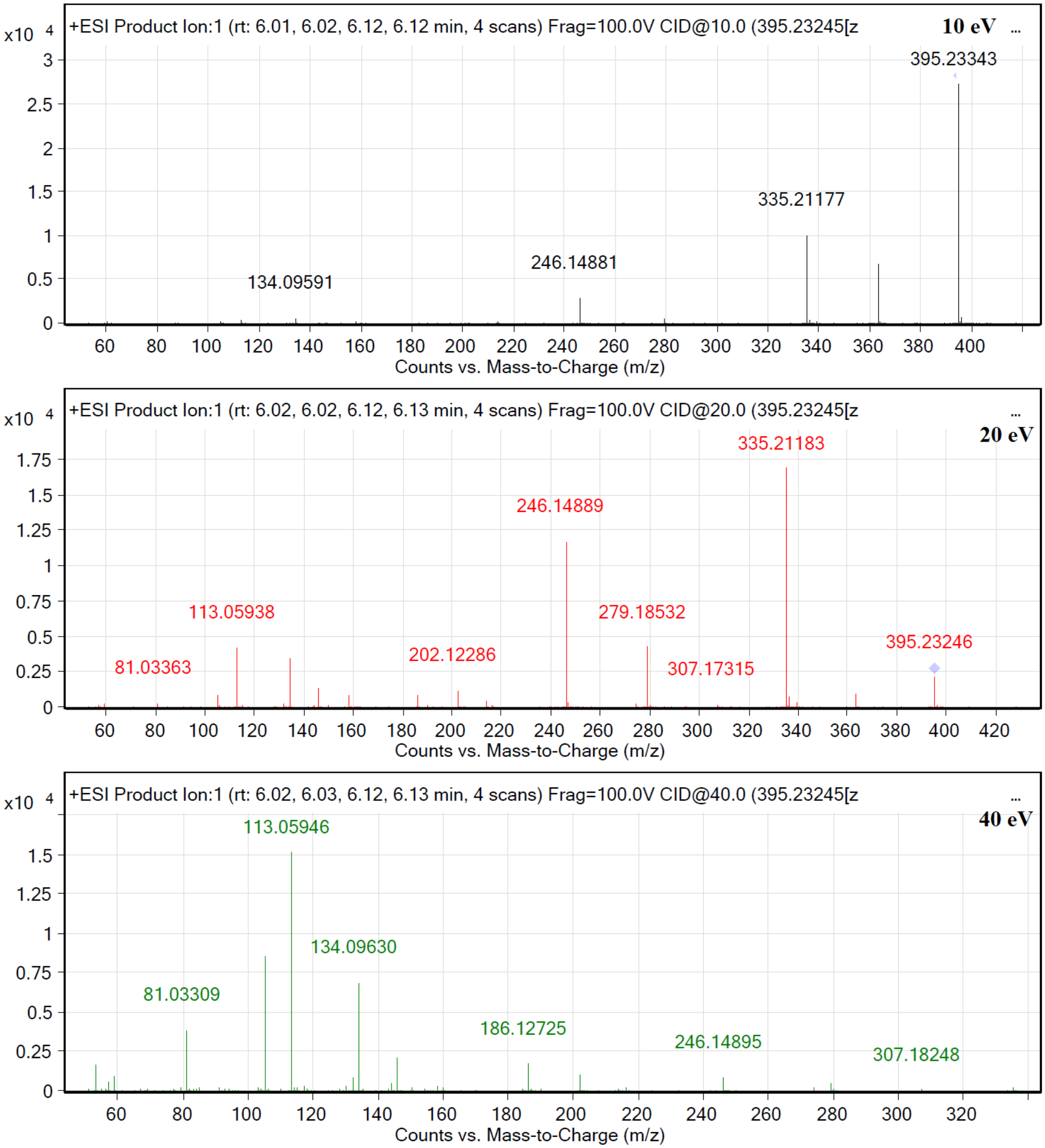

Figure S17. MS/MS spectra obtained from Carfentanil

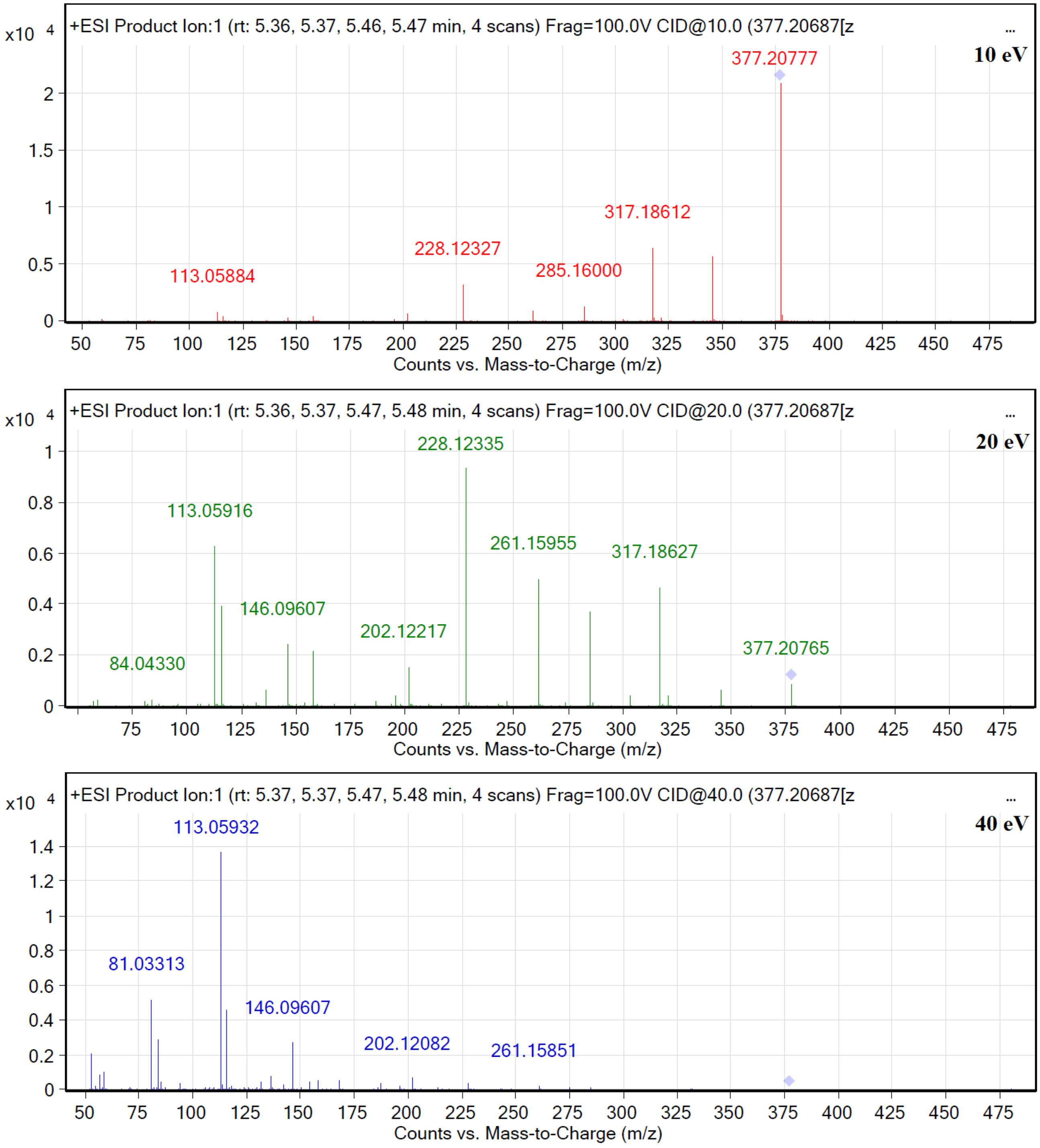

Figure S18. MS/MS spectra obtained from Remifentanil

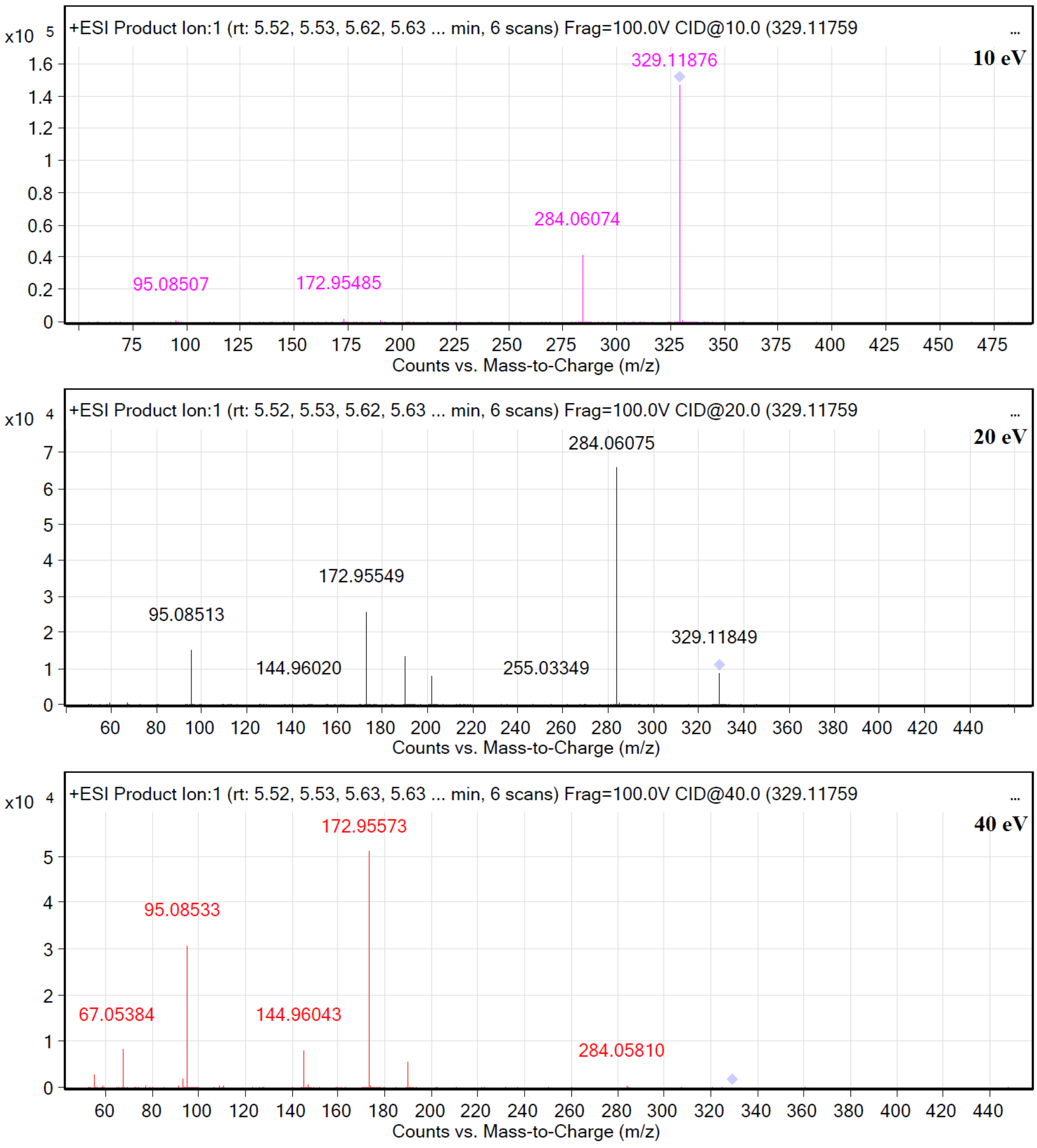

Figure S19. MS/MS spectra obtained from AH-7921

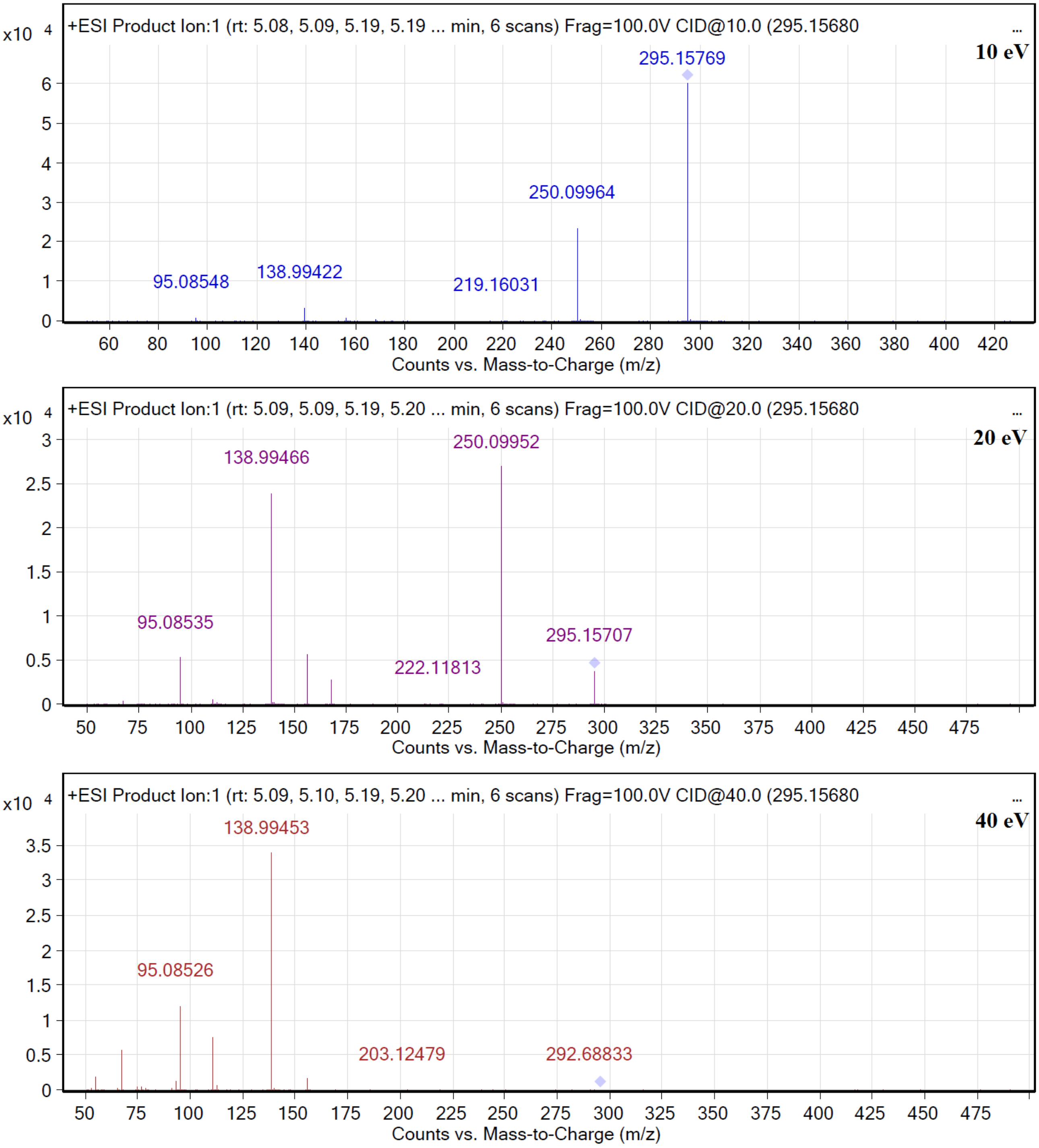

Figure S20. MS/MS spectra obtained from AH-8529

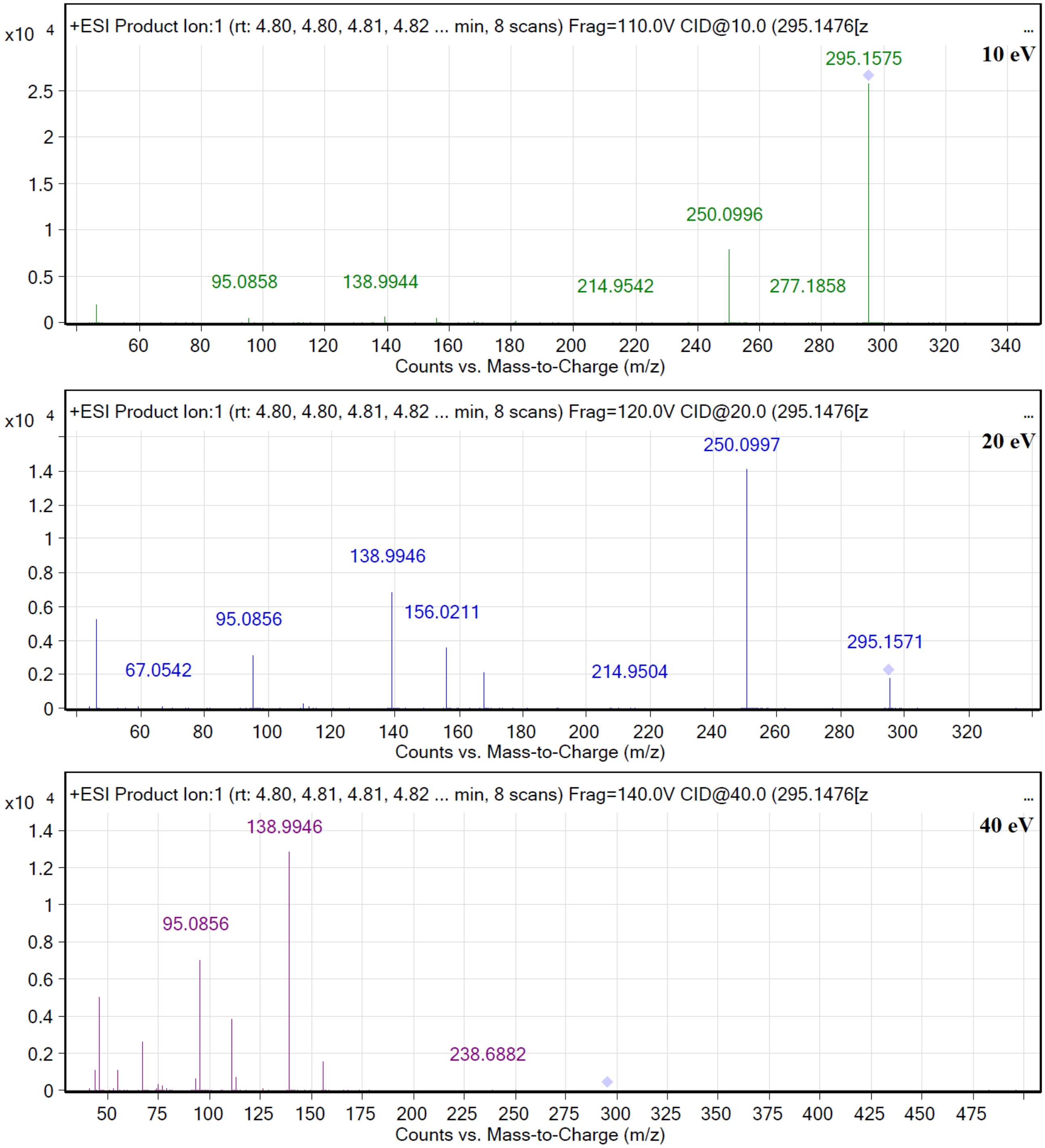

Figure S21. MS/MS spectra obtained from AH-8532

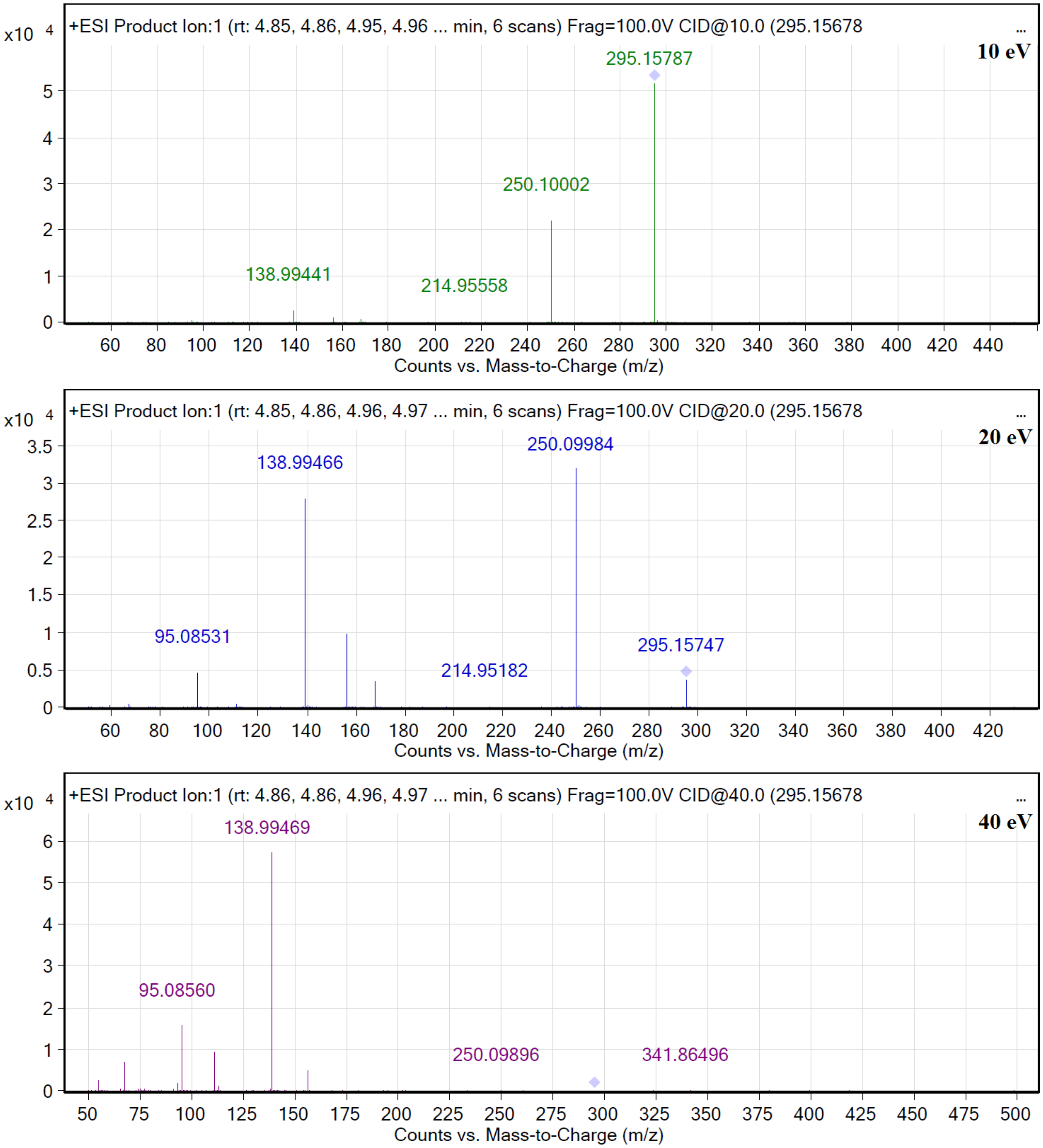

Figure S22. MS/MS spectra obtained from AH-8533

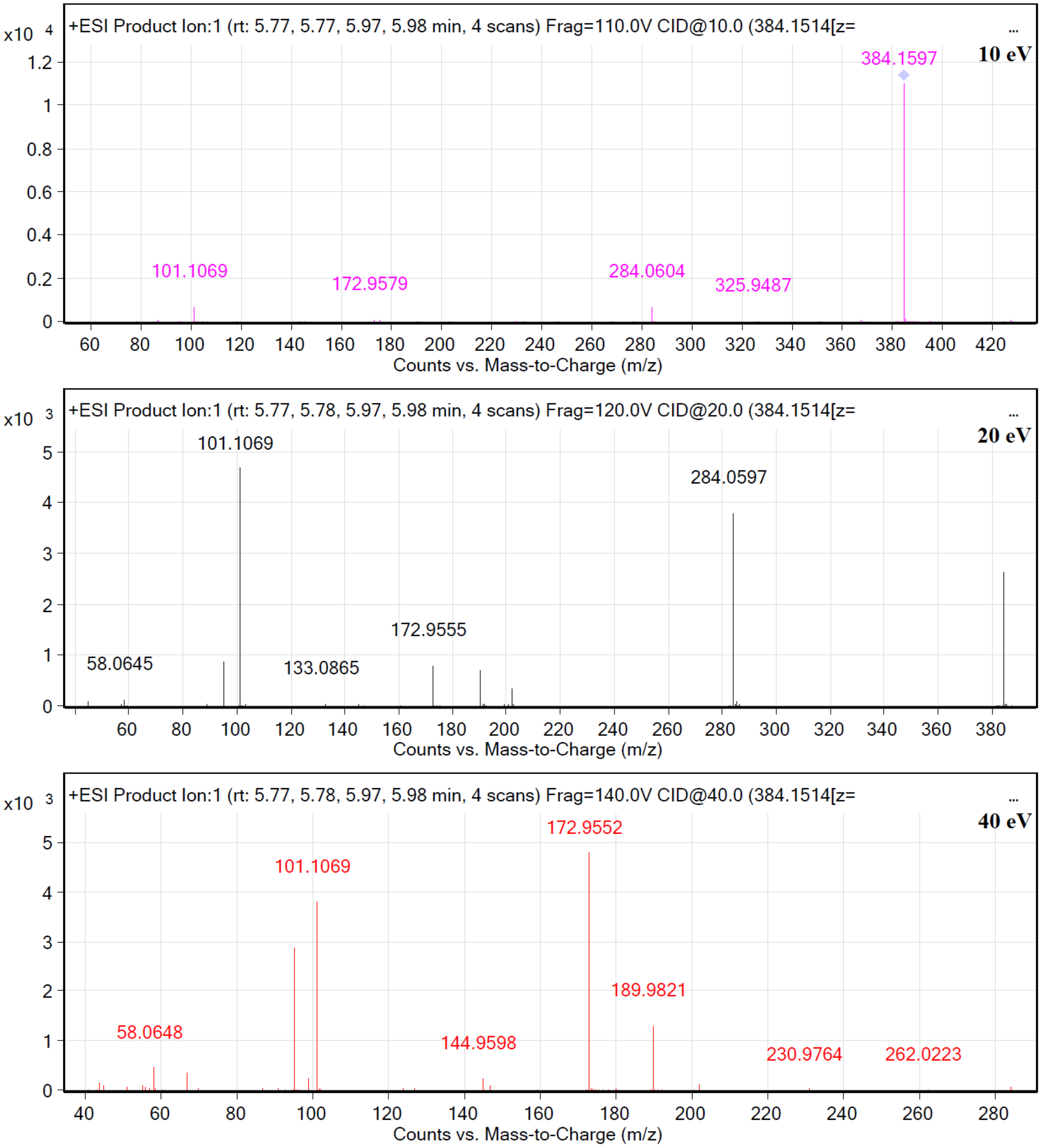

Figure S23. MS/MS spectra obtained from AH-8507

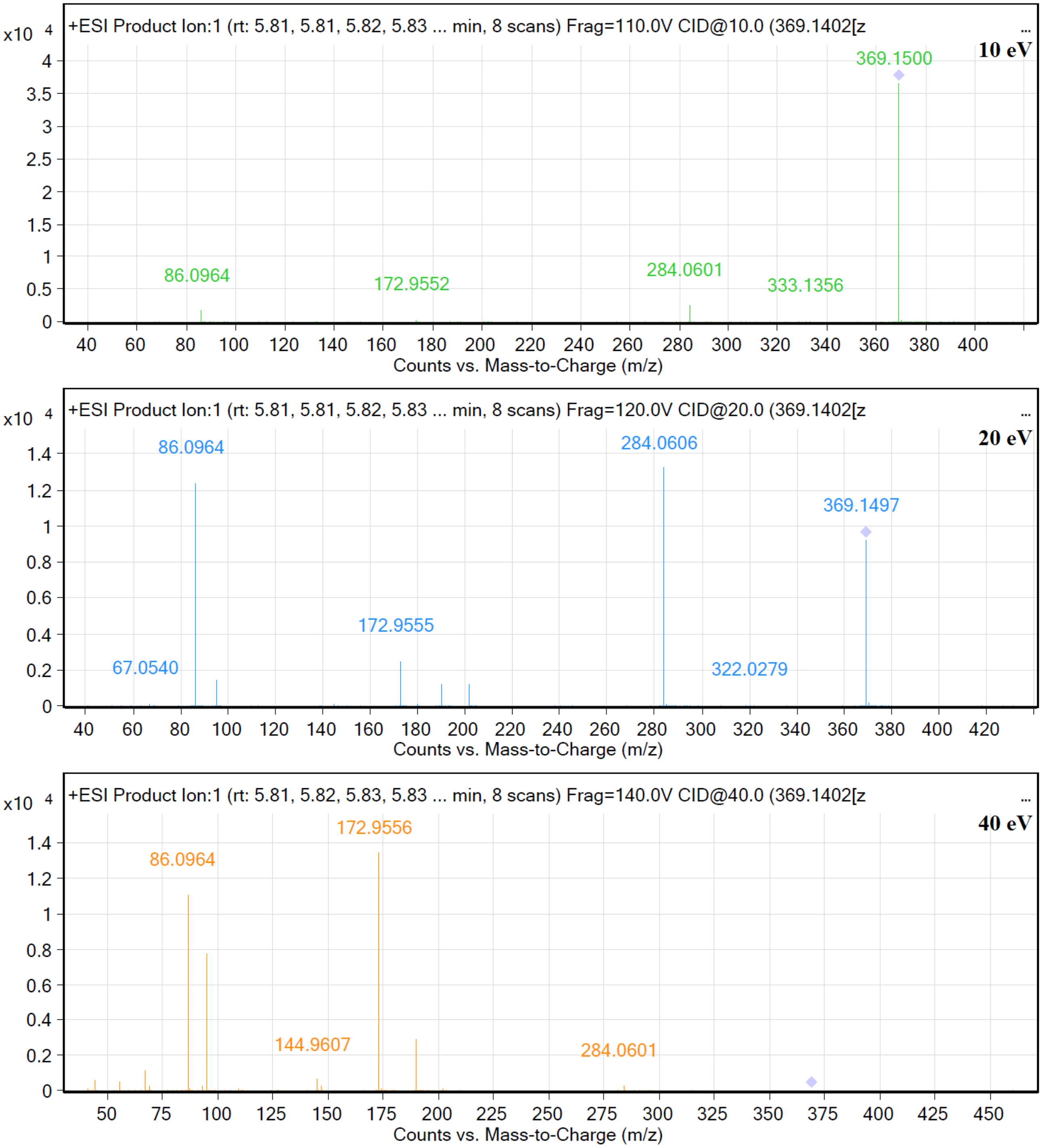

Figure S24. MS/MS spectra obtained from AH-7959

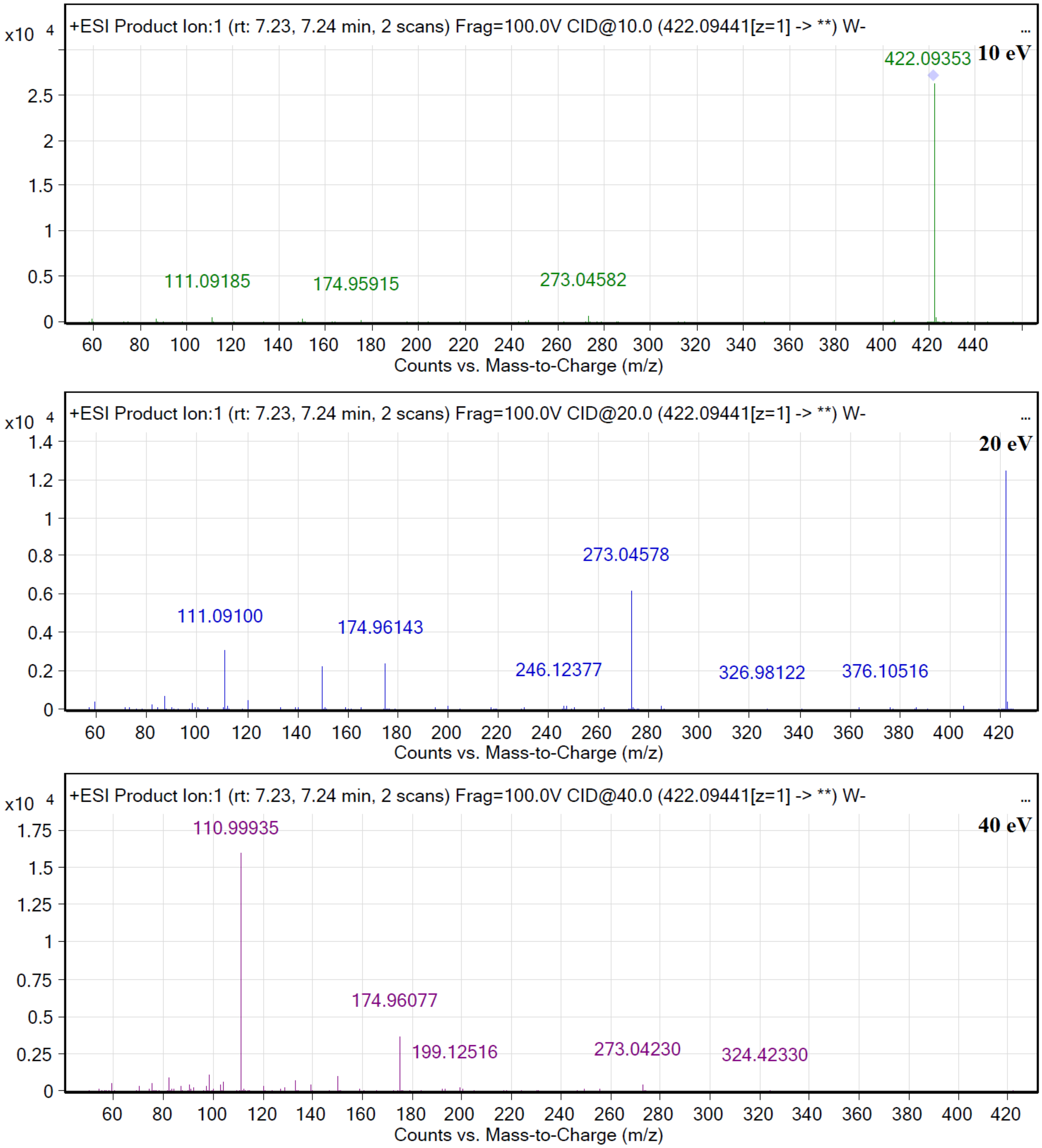

Figure S25. MS/MS spectra obtained from W-18

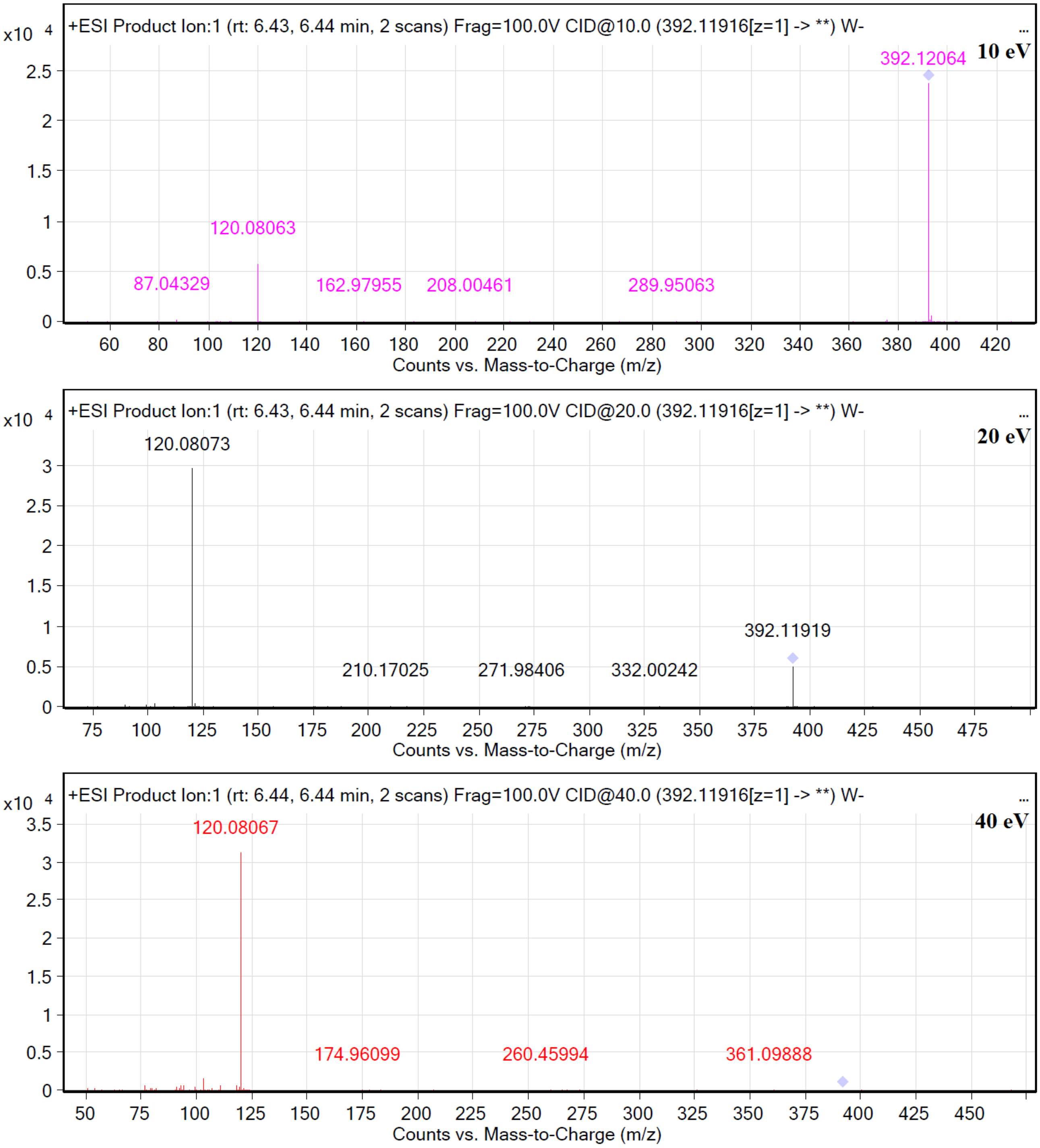

Figure S26. MS/MS spectra obtained from W-19

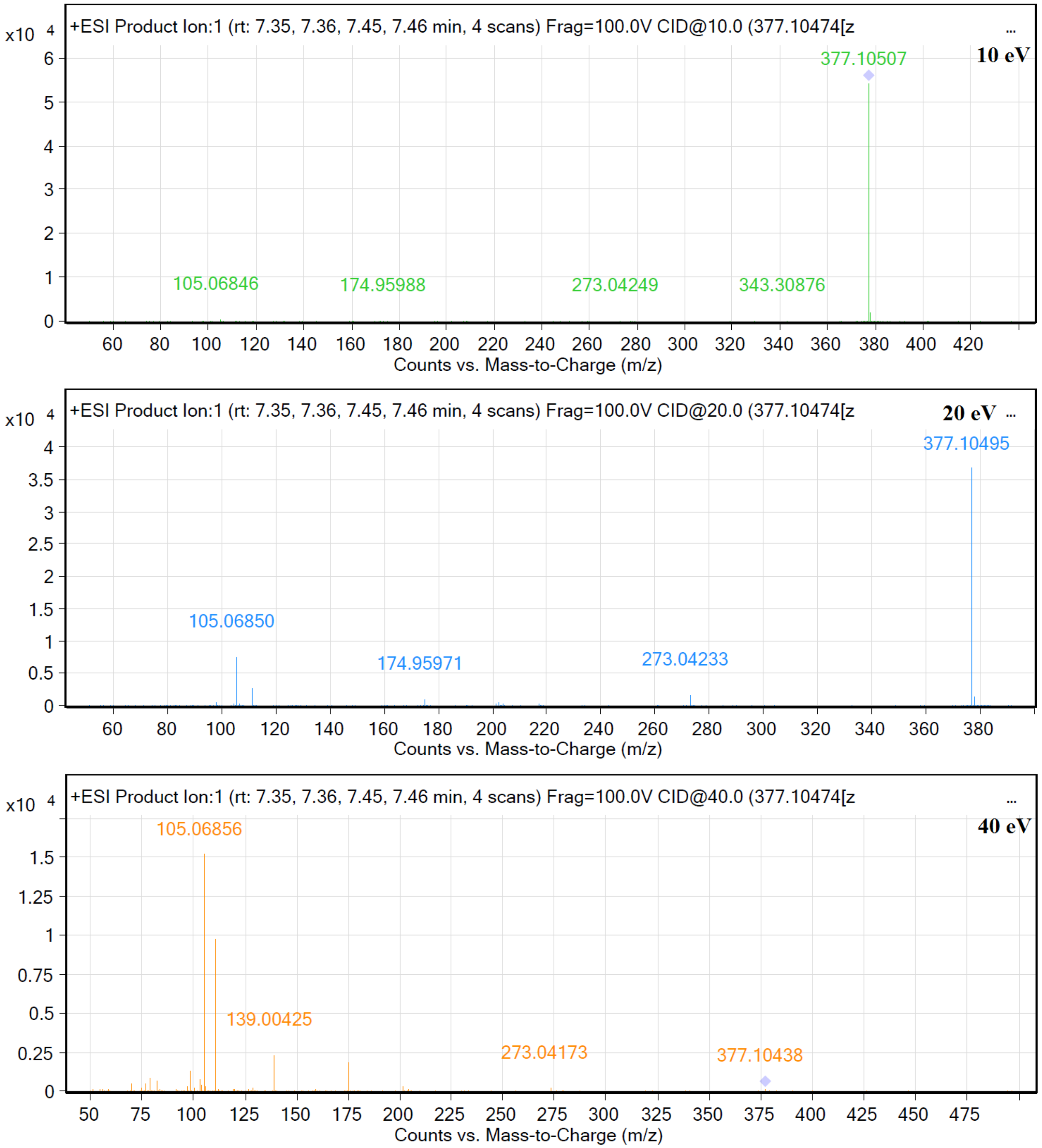

Figure S27. MS/MS spectra obtained from W-15

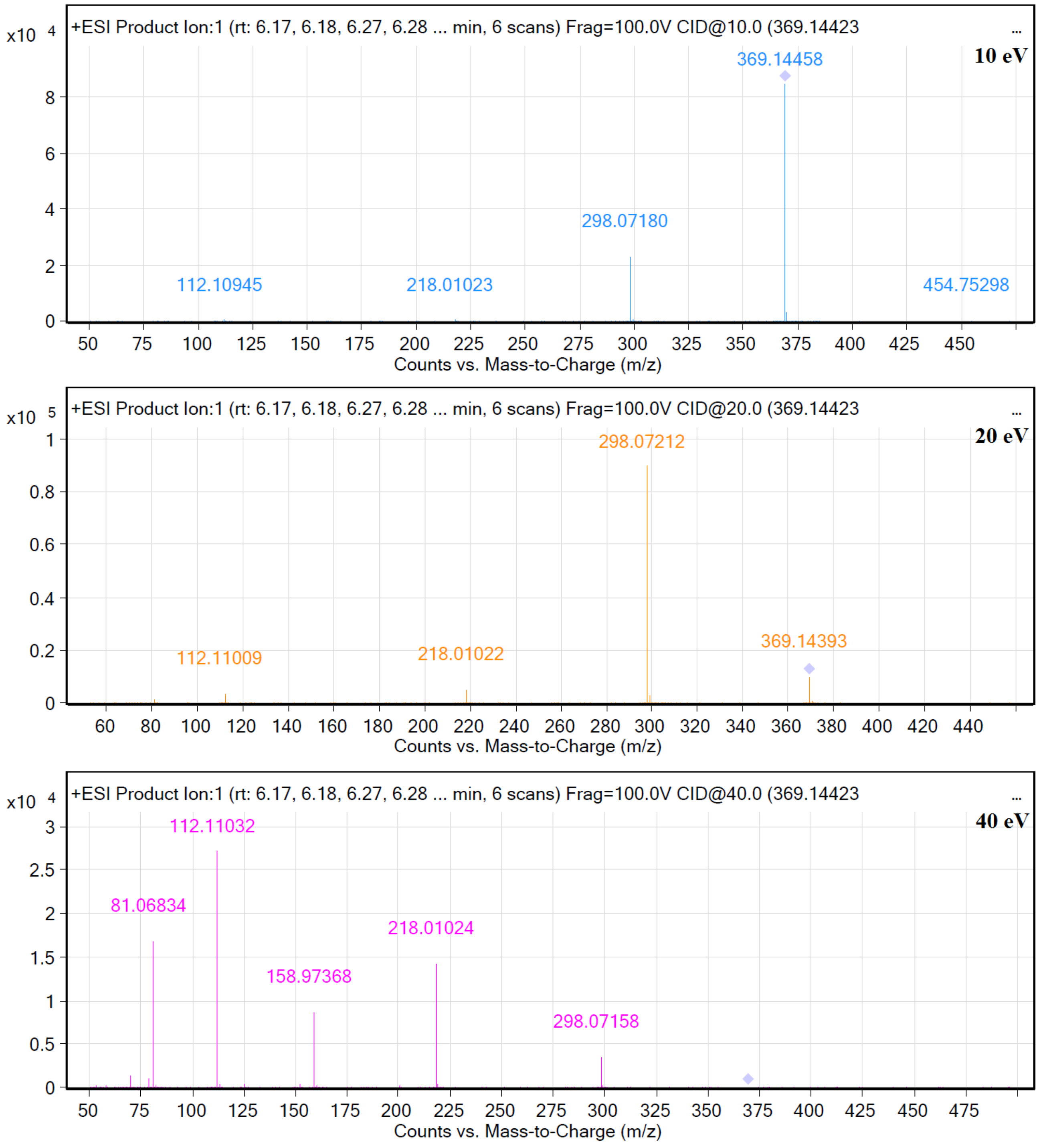

Figure S28. MS/MS spectra obtained from U-50488

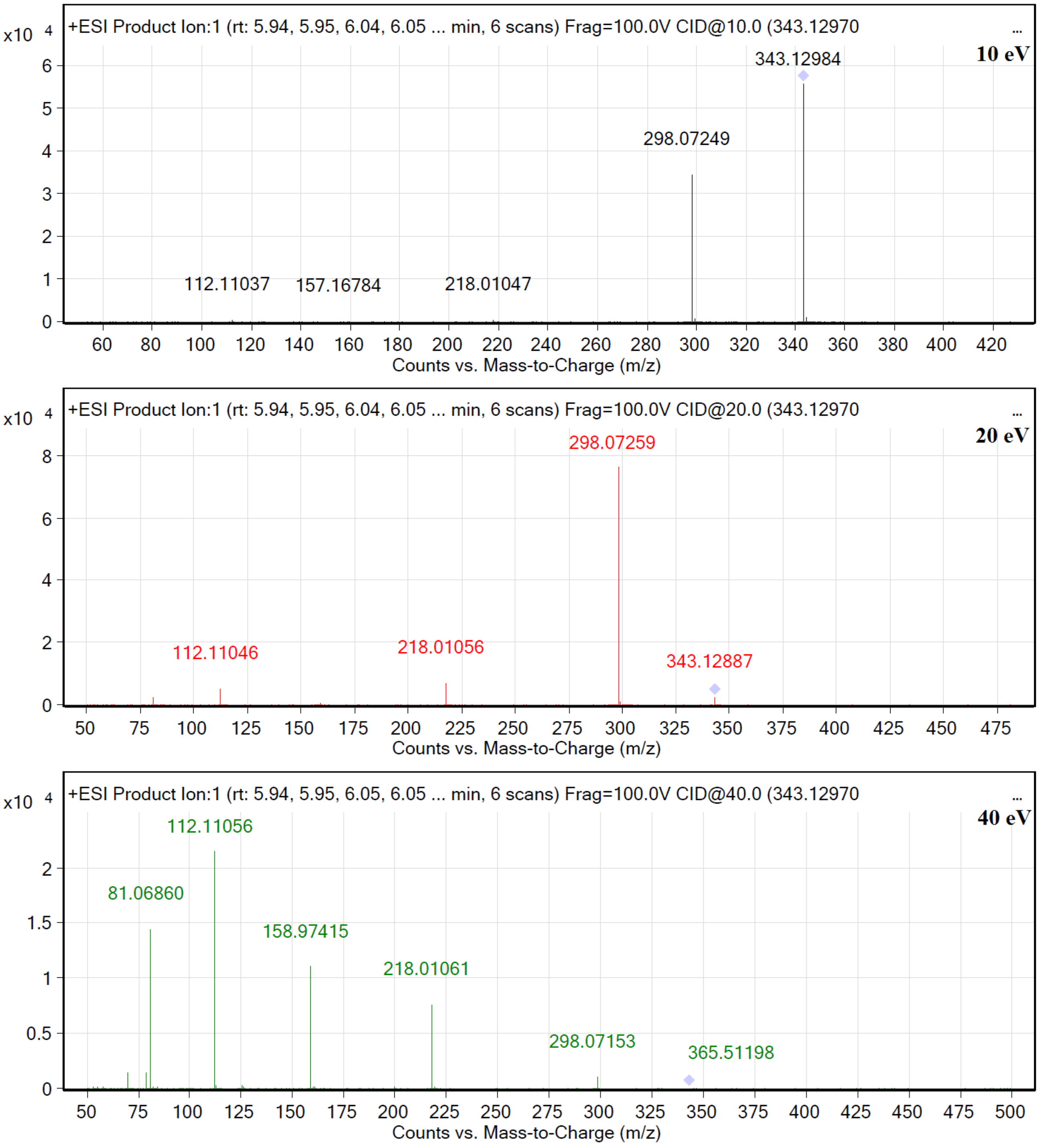

Figure S29. MS/MS spectra obtained from U-51754

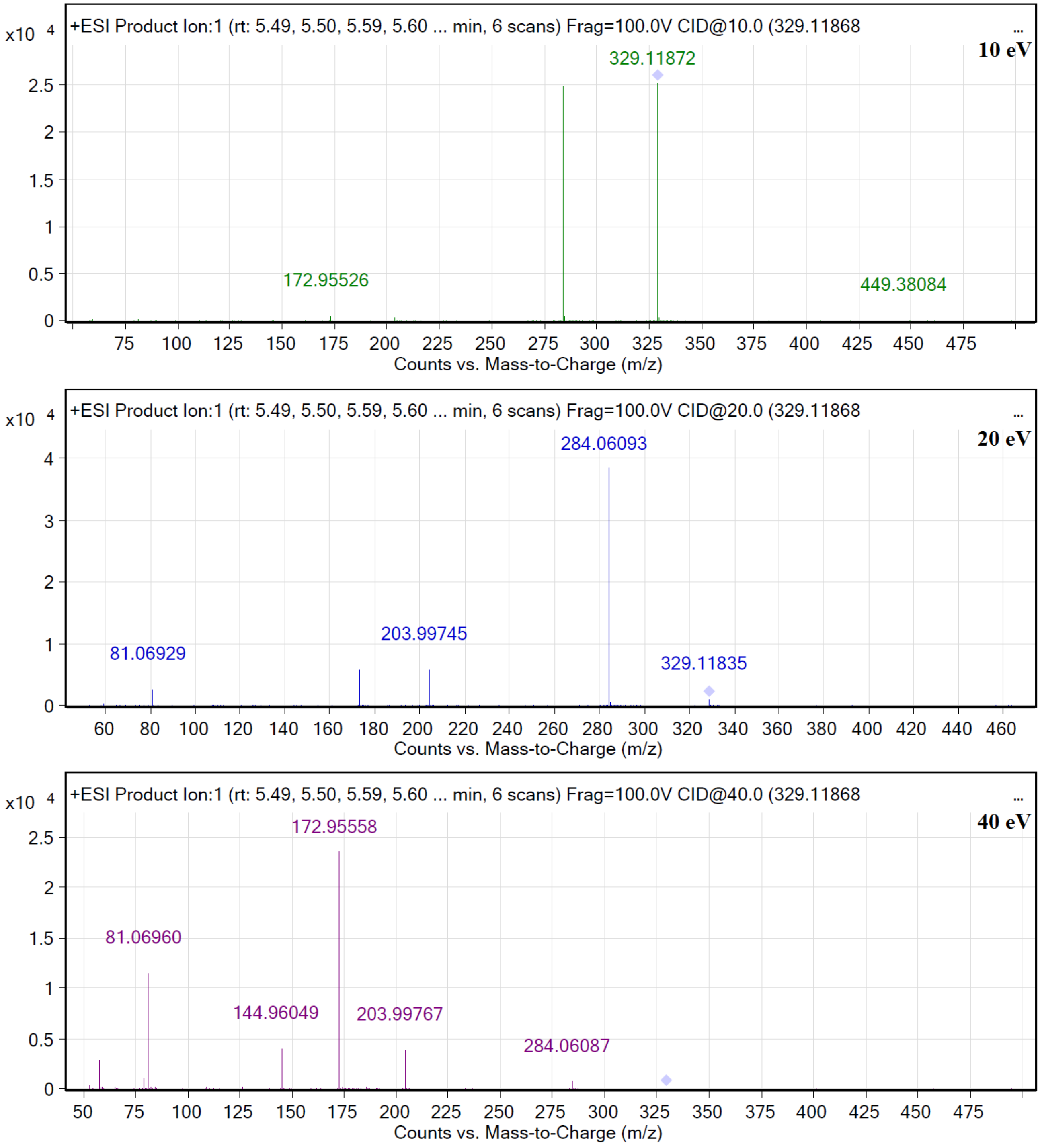

Figure S30. MS/MS spectra obtained from U-47700

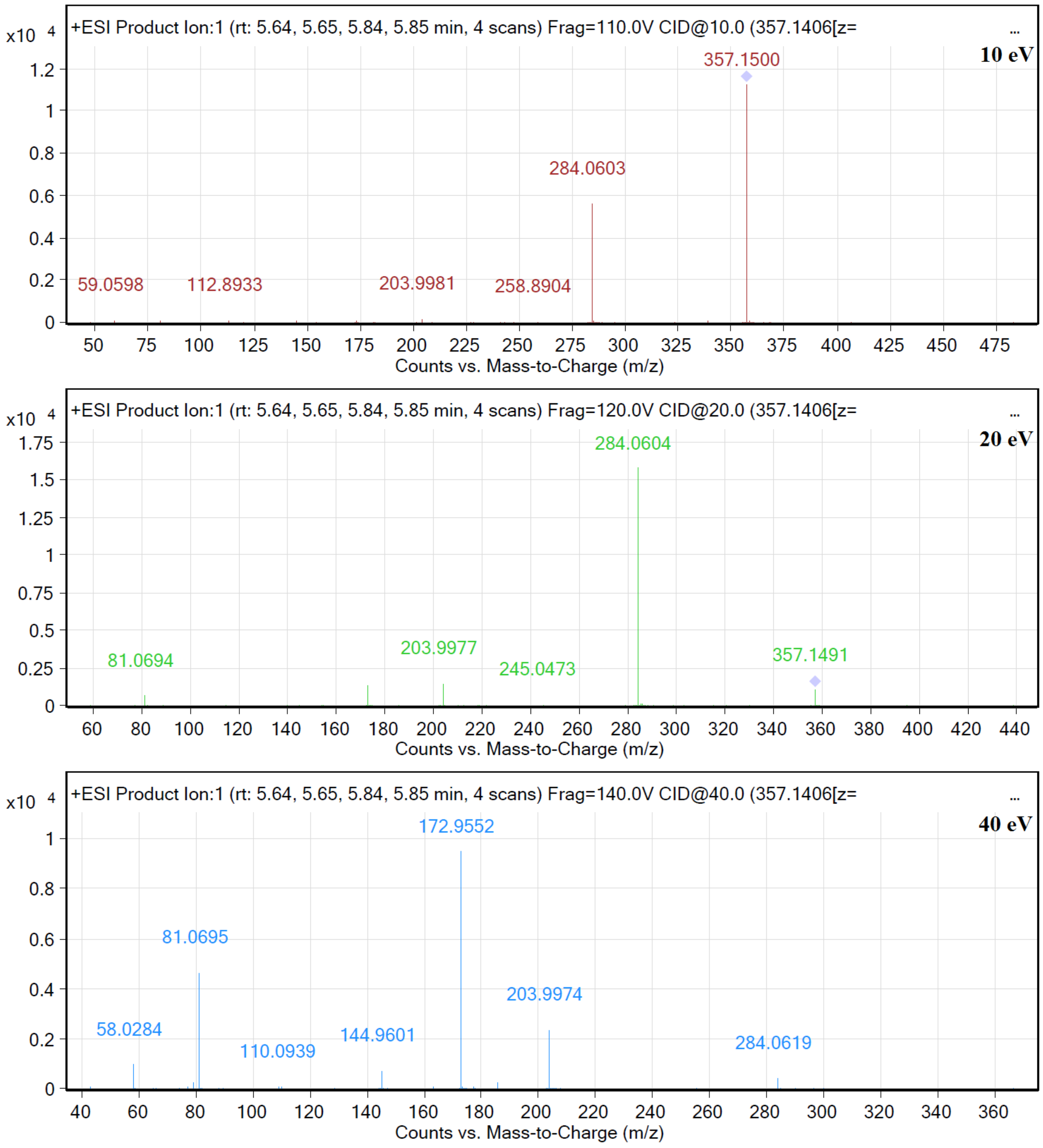

Figure S31. MS/MS spectra obtained from U-49900
